# Octahydroquinoxalin-2(1*H*)-One-Based Aminophosphonic Acids and Their Derivatives—Biological Activity towards Cancer Cells

**DOI:** 10.3390/ma13102393

**Published:** 2020-05-22

**Authors:** Jakub Iwanejko, Elżbieta Wojaczyńska, Eliza Turlej, Magdalena Maciejewska, Joanna Wietrzyk

**Affiliations:** 1 Faculty of Chemistry, Wrocław University of Science and Technology, Wybrzeże Wyspiańskiego 27, 50-370 Wrocław, Poland; jakub.iwanejko@pwr.edu.pl; 2 Department of Experimental Oncology, Hirszfeld Institute of Immunology and Experimental Therapy, Polish Academy of Sciences, Rudolfa Weigla 12, 53-114 Wrocław, Poland; eliza.turlej@hirszfeld.pl (E.T.); magdalena.maciejewska@hirszfeld.pl (M.M.); joanna.wietrzyk@hirszfeld.pl (J.W.)

**Keywords:** aminophosphonate, imine, antiproliferative activity, cell cycle, mitochondrial membrane potential

## Abstract

In the search for new antitumor agents, aminophosphonic acids and their derivatives based on octahydroquinoxalin-2(1*H*)-one scaffold were obtained and their cytotoxic properties and a mechanism of action were evaluated. Phosphonic acid and phosphonate moieties increased the antiproliferative activity in comparison to phenolic Mannich bases previously reported. Most of the obtained compounds revealed a strong antiproliferative effect against leukemia cell line (MV-4-11) with simultaneous low cytotoxicity against normal cell line (mouse fibroblasts-BALB/3T3). The most active compound was diphenyl-[(1*R*,6*R*)-3-oxo-2,5-diazabicyclo[4.4.0]dec-4-yl]phosphonate. Preliminary evaluation of the mechanism of action showed the proapoptotic effect associated with caspase 3/7 induction.

## 1. Introduction

At present, our attention is focused on the COVID-19 pandemic and there is a tendency to neglect the civilization diseases which however remain the main reason for mortality worldwide. In particular, recently published data have shown that cancers are some of the leading causes of death in Poland, with prostate cancer (almost 20% of patients) and lung cancer as the most common in the male population. The third cause of male mortality is colorectal (colon and rectum) cancer. In the female population, lung cancer dominates, followed by breast and colorectal cancer. Among young Poles, leukemias, lymphomas and brain cancers predominate and account for about 56% of cases [1].

**Figure 1:**
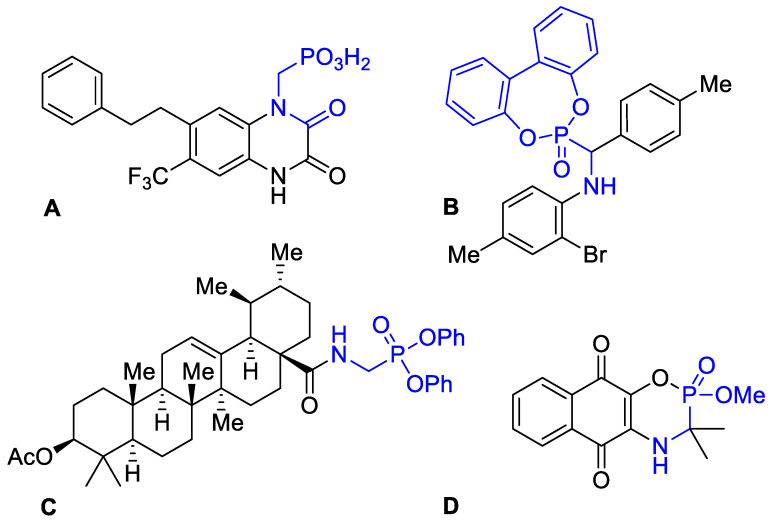
Aminophosphonic acid and aminophosphonates of potential medical importance. (**A**) AMPA (α-amino-3-hydroxy-5-methyl-4-isoxazolepropionic acid) receptor antagonist [15]; (**B**) antimicrobial aminophosphonate [16]; (**C**) anti-HIV agent [17]; (**D**) cytotoxic agent against human cervical carcinoma [18].

Anticancer drug design is a challenging field with a continuous demand for new, selective and non-toxic agents for treatment [2]. Among various classes of compounds, aminophosphonic acids and their derivatives meet these requirements. Due to their similarities to α-amino acids and a wide range of applications, α-aminophosphonic acids are continuously gaining importance in organic synthesis. So far, a number of applications such as antiviral [3,4] or cytotoxic agents [5,6], enzyme inhibitors [7,8] immune system activators [9] or antibacterial activities [10] have attracted considerable attention. The importance of these acids and their derivatives in the search for new pharmaceutical uses has been extensively discussed in numerous review articles [11,12,13,14]. The α-aminophosphonic acid and its derivatives pose a crucial role in a variety of biological activities (Figure 1), including cytotoxic properties.

Aminophosphonates bearing an *N*-heterocyclic fragment have received particular attention over the last years among other potential anticancer agents [19,20,21,22]. α-Amino acids and their esters are considered versatile pharmacophores, known for antitumor activity [23]. The group of Nikalje successfully coupled indole-2,3-diones with α-aminophosphonates, which led to new selective, antiproliferative compounds, analogues of commercially available drugs, orantinib and sunitinib [24]. In another noteworthy example, Huang and co-workers proved that the incorporation of an aminophosphonate moiety to irinotecan, a chemotherapeutic agent, increased the cytotoxicity against certain cancer cell lines [25]. The fact of their negligible mammalian toxicity [12] testifies to the usefulness of these compounds in drug discovery research.

A convenient route to this class of compounds is the Pudovik reaction—a nucleophilic addition of dialkyl phosphites to imines [26]. Another approach, Kabachnik–Fields one-pot three-component protocol, requires an amine, a carbonyl compound and an alkyl phosphite, however, it fails with electron-deficient amines [27]. Most of the reported reactions are base- or acid-catalyzed, and non-catalyzed procedures remain scarce [28]. A recent review covers the reactions and synthetic methods for aminophosphonates [29].

**Scheme 1:**
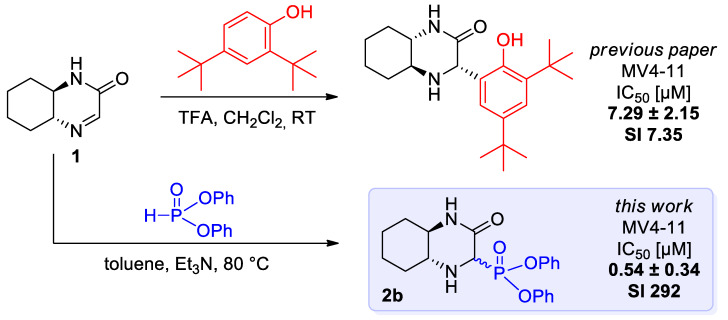
Synthesis of phenolic Mannich bases and aminophosphonates.

In our previous research, we reported on the synthesis and antiproliferative action of novel phenolic adducts of bicyclic imine 1 (Scheme 1) [30]. Two phenolic Mannich bases were found to be comparatively active to cisplatin with a noticeable increase of selectivity against cancer cell lines (one of them shown on the scheme). The tested compounds exhibit a resemblance to bioactive diketopiperazines in their bicyclic fragments. Our new goal was to synthesize phosphorus analogs of these structures and evaluate their cytotoxicity against cancer cell lines. Among a variety of synthetic methods for heterocyclic aminophosphonates formation [31], our group has focused on a convenient nucleophilic addition of dialkyl phosphites to a cyclic imine.

Bearing in mind the remarkable precedents of improving the efficacy of cytotoxicity against cancer cell lines by insertion of aminophosphonate moiety and the results of our previous research, we directed our examinations toward the evaluation of antiproliferative properties of the phosphonic derivatives of octahydroquinoxalin-2(1*H*)-one.

In our studies of the aminophosphonic acids and aminophosphonates MV4-11 (B phenotypic myelomonocytic) cell line carrying translocation t(4;11) was used. MV4-11 corresponds to AML M5b (according to the previously used French–American–British (FAB) classification based on the morphology features of the cells). Such an AML subtype is characterized by a tendency to occupy the gums, lymph nodes and skin [32]. MV4-11 cell line growth in suspension has about 50 h doubling time that makes it a good and a sensitive model for searching for new synthesized compounds against leukemia cells.

## 2. Materials and Methods

### 2.1. Compounds and General Considerations

All the reagents and solvents were purchased from commercial suppliers and used without further purification. Melting points were carried out on the Apotec^®^Schmelzpunktbestimmer melting point apparatus and are uncorrected. ^1^H, ^13^C and ^31^P NMR spectra were collected on Jeol 400yh and Bruker Avance II 600 instruments. NMR spectra were recorded in CDCl_3,_ unless specified otherwise. The temperature of the samples was 298 K. Fourier-transform infrared spectra were measured using the Perkin Elmer 2000 FTIR spectrometer. The principle peaks and their assignments are listed in Table 1. The high-resolution mass spectra (HRMS) measurements were performed using the Waters LCT Premier XE TOF instrument. Optical rotations were measured at ambient temperature on Optical Activity Ltd. Model AA-5 automatic polarimeter; [α]^D^ values are given in 10^−1^ deg cm^2^ g^−1^. Column chromatography was performed on silica gel 60 (particle size 0.063–0.200 mm). Thin-layer chromatography was conducted with the Merck silica gel 60 pre-coated plates (F_254_) and visualized with UV light and/or iodine vapors.

(1*R*,6*R*)-3-oxo-2,5-diazabicyclo[4.4.0]dec-4-ene (1). Typical procedure 

(1*R*,2*R*)-trans-diaminocyclohexane (4.00 mmol, 456 mg, 2.00 equiv) was dissolved in 2-PrOH (8 mL). To the stirred solution ethyl glyoxylate solution (50% solution in toluene, 2.00 mmol, 0.420 mL, 1.00 equiv) was added and the mixture was stirred for 24 h at room temperature (293 K). The solvent was removed in vacuo and the product was purified by silica gel column chromatography (eluent: CH_2_Cl_2_/MeOH 97:3 *v*/*v*).

Colorless solid; 273 mg, 90% yield; mp. 172–175 °C; [α]_D_^20^-238 (c 0.78, CH_2_Cl_2_); ^1^H NMR (400 MHz, CDCl_3_): δ 7.70 (t, *J* = 2.8 Hz, 1H), 7.13 (br. s, 1H), 3.05–3.14 (m, 2H), 2.34–2.36 (m, 1H), 1.76–1.95 (m, 3H), 1.31–1.45 (m, 4H); ^13^C NMR (100 MHz, CDCl_3_): δ 158.1, 156.4, 63.1, 54.2, 31.6, 31.1, 25.3, 23.7; HRMS (ESI-TOF) calcd. for C_8_H_13_N_2_O [*M*+H]^+^*m*/*z*: 153.1022 found: 153.1019.

Dimethyl-[(1R,6R)-3-oxo-2,5-diazabicyclo[4.4.0]dec-4-yl]phosphonate (2a). A typical procedure for aminophosphonates and aminophoshine oxides 2a–g 

Imine 1 (1.00 mmol, 152 mg, 1.00 equiv) was dissolved in toluene (5 mL) and appropriate H-phosphonate or diphenylphosphine oxide (1.00 mmol, 1.00 equiv) was added followed by addition of Et_3_N (1.00 mmol, 0.140 mL, 1.00 equiv). The resulting mixture was stirred for 1 h at 80 °C. After that time, the solvent was evaporated under vacuum, and the resulting crude product was purified by column chromatography (eluent: CH_2_Cl_2_/MeOH 97:3 *v*/*v*).

Light yellow solid; 189 mg; 72% yield; mixture of diastereomers, dr 56:44;^1^H NMR (400 MHz, CDCl_3_): δ 6.56 (br. s, 1H), 6.49 (br. s, 1H), 4.09 (d, *J* = 19.8 Hz, 1H), 4.01 (d, *J* = 21.7 Hz, 1H), 3.86 (d, *J* = 10.7 Hz, 3H), 3.814 (d, *J* = 11.0 Hz, 6H), 3.81 (d, *J* = 11.0 Hz, 3H), 3.05–3.11 (m, 1H), 2.96–3.02 (m, 1H), 2.84–2.90 (m, 1H), 2.40–2.46 (m, 1H), 2.08 (br. s, 2H), 1.72–1.85 (m, 8H), 1.15–1.39 (m, 8H); ^13^C NMR (100 MHz, CDCl_3_): δ 165.8, 165.6 (d, *J* = 5.5 Hz), 58.8, 58.5, 58.4, 58.1, 57.8, 57.5, 57.4, 57.0, 55.8, 55.0, 54.5 (d, *J* = 6.4 Hz), 53.9 (d, *J* = 7.3 Hz), 53.4 (t, *J* = 7.3 Hz), 30.9 (d, *J* = 16.4 Hz), 30.6 (d, *J* = 28.2 Hz), 24.4 (d, *J* = 10.9 Hz), 23.7 (d, *J* = 10.0 Hz), 23.4; ^31^P{1H} NMR (162 MHz, CDCl_3_): δ 24.3, 22.4; HRMS (ESI-TOF) calcd. for C_10_H_20_N_2_O_4_P [*M*+H]^+^ *m*/*z*: 263.1161 found: 263.1155.

Diphenyl-[(1*R*,6*R*)-3-oxo-2,5-diazabicyclo[4.4.0]dec-4-yl]phosphonate (2b)

Colorless solid; 266 mg; 69% yield; mixture of diastereomers, dr 50:50; ^1^H NMR (400 MHz, DMSO-d_6_): δ 8.15 (br. s, 1H), 8.13 (br. s, 1H), 7.31–7.37 (m, 8H), 7.14–7.20 (m, 12H), 4.34 (d, *J* = 17.7 Hz, 1H, 4*S*), 4.25 (d, *J* = 25.1 Hz, 1H, 4*R*), 2.95 (br. s, 2H), 2.83–2.89 (m, 2H), 2.58–2.63 (m, 1H, 4*R*), 2.36–2.40 (m, 1H, 4*S*), 1.77–1.80 (m, 3H), 1.59–1.63 (m, 5H), 0.98–1.28 (m, 8H); ^13^C NMR (100 MHz, DMSO-d_6_): δ 164.7 (d, *J* = 4.8 Hz), 164.3 (d, *J* = 4.8 Hz), 151.4 (d, *J* = 9.6 Hz), 150.9 (d, *J* = 3.9 Hz, 2C overlapped), 150.8 (d, *J* = 3.9 Hz, 2C overlapped), 150.7 (d, *J* = 10.1 Hz), 130.2 (4C overlapped), 130.2 (2C overlapped), 130.1 (2C overlapped), 125.6 (3C overlapped), 125.4, 121.4 (4C overlapped), 121.2 (2C overlapped), 121.1 (2C overlapped), 59.0, 58.1, 58.0, 57.3, 56.6, 56.3, 31.3, 30.8, 30.5 (d, *J* = 7.7 Hz), 25.2, 24.5, 23.9; ^31^P NMR{^1^H} (162 MHz, DMSO-d_6_): δ 15.6, 14.6; HRMS (ESI-TOF) calcd. for C_20_H_24_N_2_O_4_P [*M*+H]^+^ *m*/*z*: 387.1474 found: 387.1457.

Dibenzyl-[(1*R*,6*R*)-3-oxo-2,5-diazabicyclo[4.4.0]dec-4-yl]phosphonate (2c)

Light yellow solid; 286 mg; 69% yield; mixture of diastereomers, dr 50:50; ^1^H NMR (400 MHz, CDCl_3_): δ 7.27–7.38 (m, 20H), 6.16 (br. s, 1H), 6.10 (br. s, 1H), 5.03–5.28 (m, 8H), 4.13 (d, *J* = 19.6 Hz, 1H), 4.06 (d, *J* = 20.8 Hz, 1H), 2.94–2.99 (m, 2H), 2.82–2.88 (m, 1H), 2.35–2.42 (m, 1H), 1.87 (br. s, 2H), 1.62–1.79 (m, 8H), 1.08–1.39 (m, 8H); ^13^C NMR (100 MHz, CDCl_3_): δ 166.0, 165.9, 158.2, 156.6, 136.3–136.7 (m, 4C), 128.0–128.6 (m, 20C), 69.1 (d, *J* = 6.3 Hz), 68.5 (d, *J* = 6.7 Hz), 68.3 (d, *J* = 6.7 Hz, 2C overlapped), 59.5, 58.9, 58.4, 58.3, 57.9, 57.7, 57.6, 57.1, 54.8, 30.6 (q, *J* = 15.4 Hz), 24.4 (d, *J* = 10.6 Hz), 23.7 (d, *J* = 4.8 Hz); ^31^P NMR{^1^H} (162 MHz, CDCl_3_): δ 22.8, 20.6; HRMS (ESI-TOF) calcd. for C_22_H_28_N_2_O_4_P [*M*+H]^+^ *m*/*z*: 415.1787, found: 415.1796.

Ethyl-[(1*R*,6*R*)-3-oxo-2,5-diazabicyclo[4.4.0]dec-4-yl](phenyl)phosphinate (2d)

Colorless solid; 174 mg; 54% yield; mixture of diastereomers, dr 50:50; ^1^H NMR (400 MHz, CDCl_3_): δ 7.87–7.92 (m, 4H), 7.76–7.85 (m, 4H), 7.37–7.52 (m, 12H), 6.76 (br. s, 1H), 6.65 (br. s, 1H), 6.60 (br. s, 1H), 6.38 (br. s, 1H), 4.05–4.27 (m, 10H), 3.91–4.01 (m, 2H), 2.88–3.14 (m, 4H), 2.57–2.75 (m, 2H), 2.55 (br. s, 4H), 2.38–2.42 (m, 2H), 1.57–1.77 (m, 16H), 1.20–1.36 (m, 28H); ^13^C NMR (100 MHz, CDCl_3_): δ 166.4, 166.24, 166.20, 166.1, 133.1, 133.0, 132.9, 132.8, 132.76, 132.71 (2C overlapped), 132.64, 132.62, 132.54, 132.51, 132.48, 130.7 (2C overlapped), 130.38, 130.35, 129.5, 129.3, 129.1, 129.0, 128.6 (d, *J* = 3.9 Hz), 128.4 (d, *J* = 2.9 Hz), 128.2 (d, *J* = 6.7 Hz), 128.1 (d, *J* = 6.7 Hz), 62.2 (d, *J* = 6.7 Hz), 62.0 (d, *J* = 6.7 Hz), 61.9 (d, *J* = 6.7 Hz), 61.6, 61.1 (d, *J* = 10.6 Hz), 60.5 (2C overlapped), 60.3 (d, *J* = 10.6 Hz), 59.4, 58.5 (d, *J* = 10.6 Hz), 58.2 (d, *J* = 11.6 Hz), 57.6, 57.3 (d, *J* = 6.7 Hz), 56.9, 55.3, 55.2, 31.1, 30.9, 30.85, 30.83, 30.6 (2C overlapped), 30.5, 30.4, 24.5, 24.46, 24.42, 24.3, 23.9, 23.73, 23.70, 23.6, 16.7, 16.67, 16.63, 16.6; ^31^P NMR{^1^H} (162 MHz, CDCl_3_): δ 39.0, 38.0, 35.8, 35.3; HRMS (ESI-TOF) calcd. for C_16_H_24_N_2_O_3_P [*M*+H]^+^ *m*/*z*: 323.1525 found: 323.1526.

4-(Diphenylphosphoryl)-(1*R*,6*R*)-3-oxo-2,5-diazabicyclo[4.4.0]decane (2e)

Colorless solid; 227 mg; 64% yield; mixture of diastereomers, dr 55:45; ^1^H NMR (600 MHz, CDCl_3_): δ 8.00–8.07 (m, 4H), 7.77–7.86 (m, 4H), 7.37–7.51 (m, 12H), 7.04 (br. s, 1H), 7.02 (br. s, 1H), 4.58 (d, *J* = 13.8 Hz, 1H), 4.50 (d, *J* = 11.4 Hz, 1H), 2.90–2.94 (m, 1H), 2.85 (br. s, 2H), 2.75–2.83 (m, 1H), 2.56–2.62 (m, 1H), 2.41–2.45 (m, 1H), 1.51–1.80 (m, 8H), 1.10–1.27 (m, 8H); ^13^C NMR (151 MHz, CDCl_3_): δ 166.8 (d, *J* = 4.2 Hz), 166.7 (d, *J* = 2.1 Hz), 132.8, 132.4 (d, *J* = 9.0 Hz, 2C overlapped), 132.2 (d, *J* = 9.7 Hz, 2C overlapped), 132.0 (d, *J* = 9.7 Hz, 2C overlapped), 131.9 (2C overlapped), 131.79, 131.77, 131.6 (d, *J* = 9.7 Hz, 2C overlapped), 131.4, 131.2, 130.0, 128.5 (d, *J* = 12.5 Hz, 2C overlapped), 128.2 (d, *J* = 12.5 Hz, 2C overlapped), 128.1 (2C overlapped), 128.0 (2C overlapped), 61.6, 61.1 (d, *J* = 27.1 Hz), 60.5, 58.6 (d, *J* = 10.4 Hz), 57.1 (d, *J* = 29.1 Hz), 55.3, 30.7 (2C overlapped), 30.3 (d, *J* = 19.4 Hz, 2C overlapped), 24.4 (d, *J* = 20.1 Hz, 2C overlapped), 23.7 (d, *J* = 10.4 Hz, 2C overlapped); ^31^P NMR{^1^H} (243 MHz, CDCl_3_): δ 31.6, 29.0; HRMS (ESI-TOF) calcd. for C_20_H_24_N_2_O_2_P [*M*+H]^+^ *m*/*z*: 355.1575 found: 355.1580.

(4-((3a*R*,8a*R*)-2,2-Dimethyl-6-oxido-4,4,8,8-tetraphenyltetrahydro-[1,3]dioxolo[4,5-e][1,3,2]dioxaphosphepin-6-yl))-(1*R*,6*R*)-3-oxo-2,5-diazabicyclo[4.4.0]decane (2f)

Colorless solid; 478 mg; 72% yield; mixture of diastereomers, dr 60:40; ^1^H NMR (400 MHz, CDCl_3_): δ 7.43–7.72 (m, 16H), 7.22–7.38 (m, 24H), 7.13 (br. s, 1H, 4*R*), 5.97 (br. s, 1H, 4*S*), 5.68 (d, *J* = 8.0 Hz, 1H, 4*S*), 5.47 (d, *J* = 7.6 Hz, 1H, 4*R*), 5.38 (d, *J* = 8.0 Hz, 1H), 5.09 (d, *J* = 8.3 Hz, 1H), 4.26 (d, *J* = 18.0 Hz, 1H, 4*S*), 4.12 (d, *J* = 19.9 Hz, 1H, 4*R*), 2.96–3.00 (m, 1H, 4*S*), 2.82–2.87 (m, 2H), 2.35–2.39 (m, 1H), 2.13 (br. s, 2H), 1.58–1.70 (m, 8H), 1.21–1.33 (m, 8H), 1.07 (s, 3H), 0.77 (s, 3H), 0.47 (s, 3H), 0.38 (s, 3H); ^13^C NMR (100 MHz, CDCl_3_): δ 165.5 (d, *J* = 2.4 Hz), 165.1 (d, *J* = 7.7 Hz), 144.4 (d, *J* = 7.7 Hz), 144.2 (d, *J* = 4.8 Hz), 144.1, 143.9, 140.3 (d, *J* = 9.6 Hz), 140.1 (d, *J* = 10.6 Hz), 139.9, 139.8, 129.8, 129.4, 129.3, 129.1, 128.3, 128.25 (2C overlapped), 128.2 (2C overlapped), 128.1, 128.0 (2C overlapped), 127.9, 127.8 (2C overlapped), 127.7 (4C overlapped), 127.6, 127.5 (8C overlapped), 127.4 (8C overlapped), 127.38 (2C overlapped), 127.3 (2C overlapped), 127.2 (2C overlapped), 127.0 (2C overlapped), 114.2, 113.4, 90.7 (d, *J* = 13.5 Hz), 89.4 (d, *J* = 13.0 Hz), 88.2 (d, *J* = 9.2 Hz), 87.8 (d, *J* = 10.6 Hz), 81.0, 79.7, 79.0, 78.8, 60.1, 58.8, 58.7, 58.6, 58.3, 57.4, 55.1, 53.6, 31.0 (d, *J* = 22.2 Hz), 30.3 (d, *J* = 24.6 Hz), 27.3 (d, *J* = 14.9 Hz), 26.2 (d, *J* = 30.3 Hz), 24.5, 23.9; ^31^P NMR{^1^H} (162 MHz, CDCl_3_): δ 17.4 (4*S*), 11.3 (4*R*); HRMS (ESI-TOF) calcd. for C_39_H_42_N_2_O_6_P [*M*+H]^+^ *m*/*z*: 665.2781, found: 665.2787.

(4-((3a*R*,8a*R*)-2,2-Dimethyl-6-oxido-4,4,8,8-tetraphenyltetrahydro-[1,3]dioxolo[4,5-e][1,3,2]dioxaphosphepin-6-yl))-(1*R*,4*R*,6*R*)-3-oxo-2,5-diazabicyclo[4.4.0]decane (2g)

Colorless solid; 239 mg; 36% yield; single diastereomer separated from 2f by column chromatography (eluent: CH_2_Cl_2_/MeOH 97:3 v/v), 97% purity; mp. 176–178 °C; [α]^20^_D_ -128 (c 0.64, CH_2_Cl_2_); ^1^H NMR (400 MHz, CDCl_3_): δ 7.46–7.64 (m, 8H), 7.22–7.37 (m, 13H), 5.46 (d, *J* = 7.6 Hz, 1H), 5.36 (d, *J* = 7.6 Hz, 1H), 5.09 (d, *J* = 8.0 Hz, 1H), 4.14 (d, *J* = 20.0 Hz, 1H), 2.82–2.87 (m, 1H), 2.25–2.28 (m, 1H), 1.52–1.72 (m, 4H), 1.22–1.32 (m, 4H), 0.75 (s, 3H), 0.43 (s, 3H); ^1^C NMR (100 MHz, CDCl_3_): δ 165.5 (d, *J* = 2.4 Hz), 144.1 (d, *J* = 4.6 Hz), 143.9, 140.1 (d, *J* = 10.6 Hz), 139.7, 129.8, 129.4, 128.3 (2C overlapped), 127.9 (2C overlapped), 127.8 (2C overlapped), 127.8 (4C overlapped), 127.8 (4C overlapped), 127.3 (2C overlapped), 127.2 (2C overlapped), 127.0 (2C overlapped), 114.2, 91.1 (d, *J* = 13.5 Hz), 88.4 (d, *J* = 9.2 Hz), 79.5, 78.6, 58.6 (d, *J* = 15.5 Hz), 31.1, 29.8, 27.2, 26.4, 24.4, 23.7; ^31^P NMR{^1^H} (162 MHz, CDCl_3_): δ 10.8; HRMS (ESI-TOF) calcd. for C_39_H_41_N_2_O_6_P [*M*+H]^+^ *m*/*z*: 665.2781, found: 665.2787.

[(1*R*,6*R*)-3-oxo-2,5-diazabicyclo[4.4.0]dec-4-yl]-phosphonic acid (3a). Procedure: 

The imine 1 (1.00 mmol, 152 mg, 1.00 equiv) was dissolved in CH_2_Cl_2_ (15 mL) followed by the addition of the tris (trimethylsilyl) phosphite (1.00 mmol, 0.334 mL, 1.00 equiv). The reaction was kept at ambient temperature for 24 h, with magnetic stirring. The solvent was removed under reduced pressure and the residue dissolved in methanol (15 mL) followed by stirring overnight at ambient temperature. Methanol was removed under reduced pressure and the acid was separated by crystallization (anhydrous EtOH/Et_2_O 1:5 *v*/*v*) which led to the product as a colorless solid.

Colorless solid; 178 mg; 76% yield; mixture of diastereomers, dr 70:30; ^1^H NMR (600 MHz, D_2_O): δ 4.10 (d, *J* = 19.2Hz, 1H), 4.06 (d, *J* = 18.9Hz, 1H), 3.35–3.32 (m, 1H), 3.29–3.27 (m, 1H), 3.04–2.99 (m, 1H), 2.04–2.02 (m, 1H), 1.96–85 (m, 4H), 1.71–1.69 (m, 2H), 1.63–1.60 (m, 2H), 1.43–1.18 (m, 8H); ^13^C NMR (151 MHz, D_2_O): δ 164.9 (d, *J* = 3.5Hz), 164.5 (d, *J* = 3.4Hz), 57.5, 54.9, 53.0 (d, *J* = 121Hz), 30.3, 26.7, 23.5, 22.4; ^31^P NMR{^1^H} (243 MHz, D_2_O): δ 5.3, 5.0; HRMS (ESI-TOF) calcd. for C_8_H_16_N_2_O_4_P [*M*+H]^+^ *m*/*z*: 235.0848; found: 235.0851.

[4-phenyl-(1*R*,6*R*)-3-oxo-2,5-diazabicyclo[4.4.0]dec-4-yl]-phosphonic acid (3b). Procedure:

4-phenyl-(1*R*,6*R*)-3-oxo-2,5-diazabicyclo[4.4.0]dec-4-ene (1.00 mmol, 130 mg, 1.00 equiv) was dissolved in CH_2_Cl_2_ (10 mL) followed by the addition of the bromotrimethylsilane (1.10 mmol, 0.145 mL, 1.10 equiv) and tris (trimethylsilyl) phosphite (1.10 mmol, 0.367 mL, 1.10 equiv). The reaction mixture was flushed with argon and kept for 120 h at ambient temperature with magnetic stirring. The solvent was removed under reduced pressure and the residue dissolved in methanol (15 mL) followed by stirring overnight at ambient temperature. Methanol was removed under reduced pressure and the acid was separated by crystallization (EtOH/Et_2_O 1:5 *v*/*v*) to yield the solid product.

Colorless solid; 248 mg; 80% yield in a diastereomeric ratio >98:2 (diastereomeric ratio of crude product 85:15); mp. 196–198 °C; [α]^20^_D_ -35 (c 0.29, H_2_O); ^1^H NMR (600 MHz, D_2_O): δ 7.36–7.55 (m, 5H), 3.63–3.66 (m, 1H), 3.40–3.45 (m, 1H), 1.99–2.10 (m, 2H), 1.68–1.76 (m, 2H), 0.87–1.40 (m, 4H); ^13^C NMR (151 MHz, D_2_O): δ 166.7, 133.8 129.5, 129.1, 128.3, 69.7 (d, *J* = 185.8 Hz), 55.8, 53.0, 30.3, 27.0, 23.5, 22.5; ^31^P{^1^H} NMR (243 MHz, D_2_O): δ 8.5; HRMS (ESI-TOF) calcd. for C_14_H_20_N_2_O_4_P [*M*+H]^+^*m*/*z*: 311.1161, found: 311.1155.

The FTIR analysis was conducted to additionally confirm the structures of the products. In the spectrum of imine 1, the strong band at 1622 cm^−1^ has been assigned to the double-bonded imino group [33]. This stretching vibration was not present in the other products which confirmed the addition of phosphorus nucleophiles. Moreover, these compounds exhibited signals in the range of 1416–1453 cm^−1^ (C–N), characteristic for secondary cyclic amines, which corresponds to the spectra of aminophosphonates known in the literature [34]. Absorption in the region of 3100–3400 cm^−1^ has been attributed to the N–H stretching from the lactam group. The differences are observed for acids 3a and 3b, due to the possible intermolecular hydrogen bond formation. The P=O stretch was found at 1166–1349 cm^−1^, in accordance to the literature [35]. The other essential signals between 909 and 1122 cm^−1^ were assigned to P–O and P–Ar bonds as identified in Tusek–Bozic’s work [36].

### 2.2. Biological Activity Analysis

#### 2.2.1. Cell Culture

For evaluation of antiproliferative activity of the obtained compounds against human cancer cell lines: biphenotypic B cell myelomonocytic leukemia (MV4-11), human colon adenocarcinoma (LoVo), breast cancer (MCF-7), and lung cancer (A549) were used. Moreover, the mouse normal fibroblasts cell line (BALB/3T3) was used. The BALB/3T3, LoVo and the A549 cell lines were purchased from the ATCC (American Type Culture Collection, Rockville, MD, USA), the MV4-11 cell line was obtained from DSMZ (Leibniz Institute—German Collection of Microorganisms and Cell Culture, Braunschweig, Germany), the MCF-7 cell line from EACC (The European Collection of Cell Cultures). All the cell lines were maintained at the Hirszfeld Institute of Immunology and Experimental Therapy (IIET) in Wrocław, Poland.

The MV4-11 cell line was cultured in RPMI 1640/GlutaMax I medium (Gibco, Scotland, UK) with 10% fetal bovine serum (FBS) (HyClone Laboratories, Logan, UT, USA) and 1 mM sodium pyruvate (Sigma-Aldrich Chemie GmbH, Steinheim, Germany). The A549 cell line was cultured in the mixture of OptiMEM and RPMI 1640 (1:1) medium (both from Gibco, Scotland, UK)), with 5% FBS (HyClone Laboratories, Logan, UT, USA)), 2 mM l-glutamine, 1 mM sodium pyruvate (Sigma-Aldrich Chemie GmbH, Steinheim, Germany), while the MCF-7 cell line in Eagle’s medium (IIET, PAS, Wroclaw, Poland), 10% of FBS and 2 mM l-glutamine and 10% FBS. The medium was supplemented with 0.8 mg/L of insulin (Sigma-Aldrich Chemie GmbH, Steinheim, Germany).LoVo cell line was cultured in F12K medium (American Type Culture Collection, Rockville, MD, USA-ATCC)), with 5% FBS (HyClone Laboratories, Logan, UT, USA). BALB/3T3 cell line was cultured in DMEM (Gibco, Scotland, UK) supplemented with 2 mM l-glutamine and 5% FBS). All the culture media contained antibiotics: 100 U/mL penicillin (Polfa Tarchomin SA, Warsaw, Poland) and 100 µg/mL streptomycin (Sigma-Aldrich Chemie GmbH, Steinheim, Germany)). All the cell lines were cultured in a humid atmosphere at 37 °C and in 5% CO_2_.

#### 2.2.2. The Anti-Proliferative Assays In Vitro

Twenty four hours before adding the tested compounds, each of the cell lines was seeded in 96-well plastic plates (Sarstedt, Numbrecht, Germany) in an appropriate medium at a density (10^4^ cells/well), except A549 cell line (0.25 × 10^4^/well), and MCF7 cell line: (0.75 × 10^4^/well). The selected cell lines were exposed to each of the tested chemical compounds at four different concentrations in the range of 100 to 0.1 µg/mL for 72 h. As a reference, cisplatin (Teva Pharmaceuticals, Poland) was used, and DMSO (Sigma-Aldrich Chemie GmbH, Steinheim, Germany) served as a solvent control at concentrations corresponding to these present in the dilutions of the tested compounds. For adherent cells, a sulforhodamine B assay (SRB), and for leukemic—an MTT assay was performed.

#### 2.2.3. SRB Cytotoxic Test

After 72 h of incubation, cells were fixed in situ by gently adding of 50 µL per well of ice-cold 50% TCA (trichloroacetic acid, POCh, Gliwice, Poland) and were incubated at 4 °C for one hour. Afterwards, wells were washed five times with water and 50 µL of 0.4% solution of SRB (sulforhodamine B, Sigma-Aldrich Chemie GmbH, Steinheim, Germany) in 1% acetic acid (POCh, Gliwice, Poland) was added to each well and plates were again incubated at RT for 30 min. The unbound dye was removed by washing plates five times with 1% acetic acid, while stained cells were treated with 10 mM TRIS (Tris base, Sigma-Aldrich, Chemie GmbH, Steinheim, Germany). The absorbance in each well was read using the Elisa plate reader (BioTek Synergy H4, Swindon, UK) equipped with Gen5 software at the 540 nm wavelength [37].

#### 2.2.4. MTT Cytotoxic Test

The percentage of proliferation inhibition of leukemia cells by the tested compounds was determined by an MTT assay. Briefly, 20 µL of 3-(4,5-dimethylthiazol-2-yl)-2,5-diphenyl tetrazolium bromide solution (Sigma-Aldrich, Chemie GmbH, Steinheim, Germany) was added to each well and plates were left for 4 h at 37 °C. Then, plates were centrifuged for 5 min, at 88× *g*, at 4 °C, the supernatant was thrown out and 200 µL of DMSO per well (POCh, Gliwice, Poland) was added. The plates were left in RT for 10 min and the absorbance in each well was read using the Elisa plate reader (BioTek Synergy H4, Swindon, UK), equipped with Gen5 software at the 570 nm wavelength [38].

The results are presented as mean IC_50_ values (the concentration of the compound, that inhibits cell proliferation by 50%) ± standard deviation. IC_50_ values were assessed by the Prolab-3 system based on Cheburator 0.4, software developed by Nevozhay [39]. At each concentration, chemical compounds were tested in triplicates in a single experiment. Each experiment was repeated at least three times independently.

#### 2.2.5. Cell Cycle Distribution Analysis

For the cell cycle analysis, MV4-11 cell line was used. The cells were seeded in 24-well plastic plates (Sarstedt, Darmstadt, Germany) at a density of 0.25 × 10^6^ cells/1 mL. Next, after 24 h, the tested compounds at the final concentration IC_50_ and 2 × IC_50_, were added in a volume of 1 mL to the cells. The cells were exposed to the tested compounds for 48 h. Next, the cells growing in suspension were collected, counted with the Trypan blue solution (Sigma-Aldrich Chemie GmbH, Steinheim, Germany), centrifuged for 5 min at +4 °C, 5 min, at 324× *g*, resuspended in 1 mL of 70% ice-cold ethanol (POCH, Gliwice, Poland) and frozen at −20 °C for at least 24 h. After that, the cells were transferred to 5 mL propylene tubes (dedicated for flow cytometry analysis), washed in PBS (IIET, PAS, Wroclaw, Poland) and centrifuged (+4 °C, 10 min, 324× *g*). Then, the RNase solution (in PBS, 8 µg/mL) (Life Technologies, Carlsbad, CA, USA) was added (500 µL for 0.5 × 10^6^ cells) and the cells were incubated for 60 min at 37 °C with gentle mixing. After that, the cells were placed on ice, a propidium iodide (PI) solution (Sigma-Aldrich Chemie GmbH, Steinheim, Germany) (in concentration 0.1 mg/mL) was added to the cells for 30 min. Next, the flow cytometry analysis of the cell cycle was performed using BD LSR II Fortessa (Becton Dickinson, San Jose, CA, USA), equipped with FACS Diva version 6.1. Software (BD). The analysis of the obtained results was performed using Flowing Software version 2.5.1 developed by Perttu Terho. For each sample, the percentage of cells in each cell cycle phase was determined. Each experiment was performed three times independently.

#### 2.2.6. Apoptosis vs. Necrosis Determination by Annexin V/Propidine Iodine Staining

The MV-4-11 cell line was seeded at the density of 0.25 × 10^6^ cells/mL (total 1 × 10^6^ cells) in a culture medium on 24-well plastic plates (Sarstedt, Numbrecht, Germany) and was exposed to the chemical compounds at the concentration of IC_50_ and 2 × IC_50_ for 48 h. As a solvent control, DMSO (POCh, Gliwice, Poland) was used at a concentration corresponding to the highest concentration of compounds. After 48 h of incubation, the cells were collected, washed in PBS (324 g, 10 min, 4 °C) and counted.

The cells (0.5 × 10^6^/mL) were diluted in a 0.5 mL annexin binding buffer (10 mM HEPES/NaOH; 140 mM NaCl, 2.5 mM CaCl_2_: IIET PAS, Wrocław, Poland), diluted in distilled water at a ratio of 1:4. 5 µL of annexin V conjugated with APC (BD Bioscience, San Jose, CA, USA) was added to each 195 μL of cell suspension. After 15 min of incubation at room temperature in the dark, and PBS washing, the PI solution at 0.5 mg/mL (Sigma-Aldrich GmbH Chemie, Steinheim, Germany) was added to the samples. The data were processed using BD LSRII Fortessa, equipped with Diva 6.1 software. The data were analyzed using Flowing Software version 2.5.1., developed by Perttu Terho and described as: double negative (live), double-positive (late apoptotic), annexin V positive—propidine iodine negative (early apoptotic)—and annexin V negative—propidine iodine positive (necrotic). Each experiment was repeated 4–5 times.

#### 2.2.7. Caspase-3 Activity Detection

The MV-4-11 cells were seeded at the density of 0.25 × 10^6^ cells/mL in culture medium on 24-well plastic plates (Sarstedt, Numbrecht, Germany). After a 48 h exposition to the compounds at the concentration of IC_50_ and 2 × IC_50_ and incubated for 48 h, cells were collected and washed in PBS. As a solvent control, DMSO was used at the concentration corresponding to the highest concentration of the compounds. Camptothecin (Sigma-Aldrich GmbH Chemie, Steinheim, Germany) was used as a positive control.

The appropriate volume of lysis buffer pH 7.5 (50 mM HEPES, 10% sucrose, 150 mM NaCl, 1% Triton X-100) (IIET, PAS, Wroclaw, Poland) with the addition of 1% of DTT (DL-Dithiotreithol, Sigma-Aldrich GmbH Chemie Steinheim, Germany) was prepared. The reaction buffer pH 7.5 (20 mM HEPES, 10% sucrose, 100 mM NaCl) IIET PAS, Wrocław, Poland with the DTT addition and with 10 µM of caspase-3 substrate (Ac-DEVD-AMC, Cayman Chemicals, Ann Arbor, MI, USA) was prepared and warmed to 37 °C before using.

After incubation time the cells were centrifuged (324× *g*, 10 min, 4 °C), 50 μL of the lysis buffer was added to each sample and the probes were left at 4 °C for 30 min. Then 40 μL of lysates were added to white 96-well plates (Perkin-Elmer, Waltham, MA, USA) in triplicate, 160 μL of a pre-warmed reaction buffer was added to each well and the fluorescence was read out using a fluorescence plate reader (BioTek Synergy H4, Swindon, UK) equipped with Gen5 software The measurements were performed at 355 nm and 460 nm for 2 h every 10 min at 37 °C. At the same time, an MTT test was performed in order to normalize results. Based on the results obtained, an MFI (mean fluorescence intensity) vs. reaction time curve was plotted and the Vmax (reaction rate) values were determined. After normalization, the values obtained for the tested samples were compared to the control to assess how many times caspase-3 activity in tested probes is higher/lower than in control. Each experiment was conducted at least 4–5 times.

#### 2.2.8. Mitochondrial Membrane Potential Flow Cytometry Detection

The MV-4-11 cell line was seeded at the density of 0.25 × 10^6^ cells/mL in a culture medium on 24-well plates (Sarstedt, Numbrecht, Germany). The cells were exposed to the compounds at the concentration of IC_50_ and 2 × IC_50_ for 48 h. Then, the cells were collected, washed in PBS and counted in a Trypan blue solution.

The collections of 0.2 × 10^6^ cells/sample were centrifuged (300× *g*, 5 min, room temperature) and pellets were suspended in a JC-1 solution (Cayman Chemicals, Ann Arbor, MI, USA) in a warm culture medium (final concentration 2.5 µg/mL). After 10 min of incubation at 37 °C in the dark, cells were centrifuged (300× *g*, 5 min, room temperature) and pellets were suspended in 200 µL of PBS (IIET, PAS, Wrocław, Poland). As a solvent control, DMSO was used at the concentration corresponding to the highest concentration of the compounds. Valinomycin (Sigma-Aldrich GmbH Chemie, Steinheim, Germany) was used as a positive control.

The results were read using BD LSRII Fortessa (Becton Dickinson, San Jose, CA, USA), equipped with FACS Diva 6.1. software and were analyzed with Flowing Software 2.5.1, developed by Perttu Terho in dot plots presenting JC-1 monomers to aggregates.

#### 2.2.9. Dansyl Cadaverine Fluorescence Detection

The MV-4-11 cells were seeded at a density of 0.25 × 10^6^ cells/mL in culture medium on 24-well plates (Sarstedt, Numbrecht, Germany). The cells were exposed to the compounds at the concentration of IC_50_ and 2 × IC_50_ and incubated for 48 h. As a solvent control, DMSO was used at a concentration corresponding to the highest concentration of the compounds. Tamoxifen (Sigma-Aldrich GmbH Chemie, Steinheim, Germany) was used as a positive control. After 48 h of incubation, the cells were collected and washed in PBS (324 g, 10 min, 4 °C).

For this purpose, 96-well black plates were used (Perkin Elmer, Walthman, MA, USA). 100 μL of propidine iodine (PI) (final concentration: 10 µg/mL) was added to each sample, except the probes intended for background measurement. After 2 min of room temperature incubation, the cells were centrifuged (400× *g*, 5 min, room temperature), pellets were suspended in 100 µL of PBS (IIET PAS, Wrocław, Poland) and again centrifuged. Next 100 µL of 0.05 mmol/L dansyl cadaverine (Sigma Aldrich GmbH Chemie, Steinheim, Germany) was added to each sample and the samples were incubated at 37 °C for 10 min. Next, the cells were centrifuged and washed with PBS. Finally, the obtained pellet was suspended in 300 µL of PBS and transferred into the appropriate wells. Each sample was made in triplicate.

Autophagic vacuole staining intensity was detected at the excitation wavelength of 335 nm and emission of 512 nm, and the degree of cell death at excitation wavelength 536 nm and emission of 617 nm. Each experiment was conducted at least 4–5 times.

#### 2.2.10. Acridine Orange Flow Cytometry Determination

The MV-4-11 cell line was seeded at the density of 0.25 × 10^6^ cells/mL in a culture medium on 24-well plastic plates (Sarstedt, Germany) to the final volume of 2 mL. The cells were exposed to the compounds at the concentration of IC_50_ and 2IC_50_ and incubated for 48 h. As a solvent control, DMSO was used at the concentration corresponding to the highest concentration of the compounds. After 48 h of incubation, the cells were collected, washed in PBS (324 g, 5 min, room temperature) and counted.

The collection of 0.2 × 10^6^ cells were stained with 10 µg/mL of acridine orange (AO) (Sigma Aldrich GmbH Chemie, Steinheim, Germany) for 20 min at 37 °C, washed two times with PBS and read using BD LSR II Fortessa (Becton Dickinson, San Jose, CA, USA), equipped with FACS Diva 6.1. software and were analyzed with Flowing Software 2.5.1, developed by Perttu Terho. Each experiment was performed at least 4 times.

## 3. Results and Discussion

### 3.1. Chemistry

The presented compounds were synthesized using the previously published methods [40,41]. A cyclic imine 1, derived from optically pure trans-(*R*,*R*)-1,2-diaminocyclohexane was reacted with *H*-phosphonates or phosphine oxides to give compounds 2a–f with good yields as mixtures of epimers (Table 2). A single epimer 2g of *P*-TADDOL derivative (TADDOL = α,α,α′,α′-tetraaryl-2,2-disubstituted 1,3-dioxolane-4,5-dimethanol) was obtained by crystallization from mixture 2f. NMR spectra of products 1 and 2a–g are shown in the Supplementary Materials (Figures S1–S8).

**Scheme 2:**
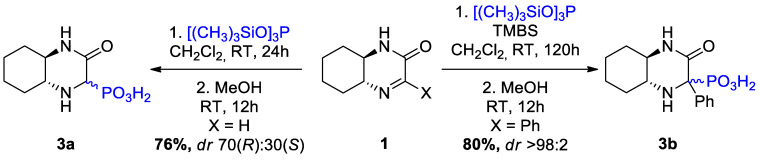
Synthesis of aminophosphonic acids (TMBS—bromotrimethylsilane).

In a modified protocol, we obtained aminophosphonic acids 3a and 3b (Scheme 2). An addition of tris(trimethylsilyl)phosphite and further methanolysis yielded product 3a (Supplementary Materials, Figure S9). When a ketimine (X = Ph) was used in the reaction, an additive of bromotrimethylsilane was required for the reaction to complete. An activation of C=N bond was necessary, since C-substituted imines are less reactive. The diastereoselectivity of the reactions was improved, especially in the case when a sterically hindered phenyl ketimine was used. The dr values provided in Table 2 represent the compositions of the epimeric mixtures used in further studies and were determined by ^31^P NMR and confirmed by ^1^H NMR spectroscopy. In the case of compound 3b (Supplementary Materials, Figure S10), only traces of the second epimer were detected at the level of the accuracy of NMR technique (ca. 2%).

### 3.2. Antiproliferative Effect of Tested Compounds

All compounds were evaluated according to their antiproliferative activity towards human acute myeloid leukemia (AML-M5b) cell line (MV4-11) and three adenocarcinoma cell lines of different origin: lung (A549), colorectal (LoVo) and breast (MCF-7). The results were compared with those obtained on the normal murine fibroblasts cell line (BALB/3T3).

Among the modified compounds, only derivatives 2f and 2g (similarly to 1) exert antiproliferative activity against all the cancer cell lines tested, however in contrast to compound 1 their activity towards BALB/3T3 cells was visibly lower as compared to the cancer cells (Table 3). On the other hand, 2b and 3a were most active towards leukemia cells, though they were not toxic neither for solid tumors cell lines nor murine fibroblasts.

A preliminary SAR (structure–activity relationship) analysis reveals a few conclusions about structural features that result in the desired activity. Among the tested phosphonates, phenyl derivative 2b performed better than benzyl (2c)**,** methyl (2a) and TADDOL esters (2f). However, the latter derivative bearing a substituent introducing a big steric hindrance was found to be more versatile and acted on all of the investigated cell lines. Comparison of diastereomeric mixture (2f) and isolated single isomer (2g) reveals no significant differences between epimers with opposite configurations of the stereogenic center C-4. Therefore, the separation of phosphonate diastereomers for biological tests seems unnecessary.

Remarkably, *H*-phosphinate 2d was practically inactive, while phosphine oxide 2e exhibited a moderate activity in comparison to the majority of the tested phosphonates. This indicates the directions of further modifications, which should be focused on esters of aminophosphonic acids. The acid itself (3a) exhibited high cytotoxicity, but only toward leukemia cells; comparison of compounds 3a and 3b suggests that complete substitution of a stereogenic center decreases the antiproliferative activity.

**Figure 2:**
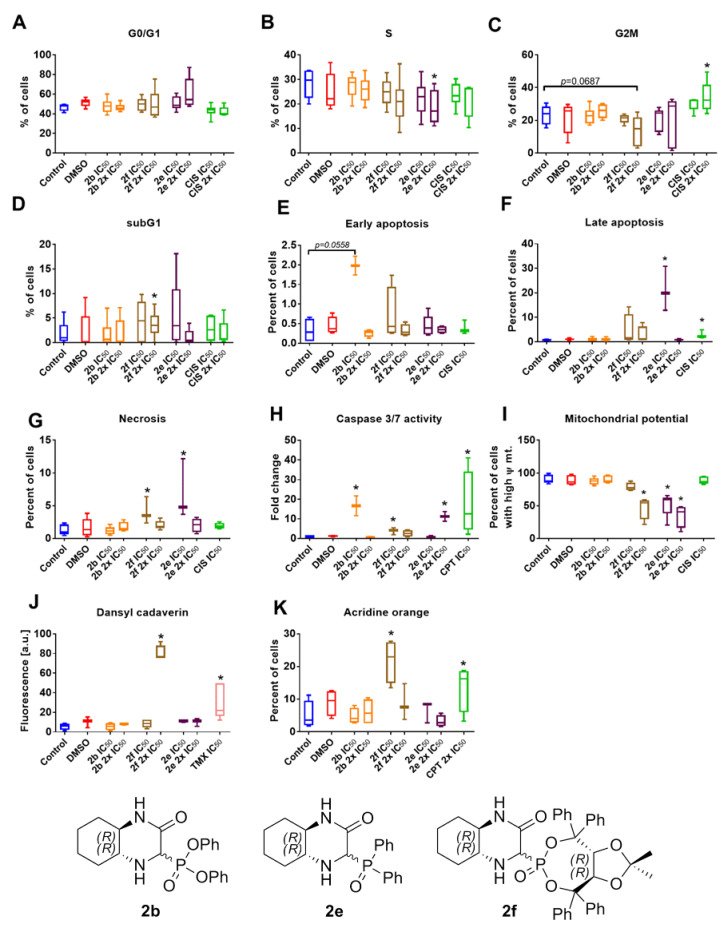
Biological activity of tested compounds on MV4-11 leukemia. (**A**–**D**) Cell cycle distribution including cells in (**A**) G0/G1, (**B**) S, (**C**) G2M and (**D**) death cells (sub G1). (**E**–**G**) Annexin V staining: (**E**) early apoptosis, (**F**) late apoptosis and (**G**) necrotic cells. (**H**) Activity of caspase 3/7 measured using Ac-DEVD-AMC caspase substrate. (**I** Mitochodrial potential measured with the use of JC-1 staining. (**J**,**K**) Analysis of autophagy using two methods with: (**J**) dansyl cadaverin and (**K**) acridine orange staining. MV4-11 cells were incubated for 48 h with two different concentrations of compound 2b, 2f and 2e: IC_50_ and 2 × IC_50_ calculated in 96 h proliferation test (MTT). As a control, selected anticancer agents were also used: cisplatin (abbreviated as CIS), camptothecin (CPT), and tamoxifen (TMX). DMSO was used in the concentration corresponding to the highest concentration of the compounds. The tests were repeated 3–5 times. Data are presented as box and whisker plot: the ends of the box are the upper and lower quartiles; the median is marked by a vertical line inside the box; the whiskers: min-max values. Statistical analysis: the Kruskal–Wallis test followed by Dunn’s multiple comparison test was performed. * *p* < 0.05, as compared to the control.

Compounds 2b, 2e and 2f were selected for further studies. For evaluation of the impact of the selected compounds on cell cycle distribution, we analyzed the percentage of cells in each of the cell divisions upon incubation with selected compounds. Analyzing cell cycle distribution (Figure 2A–D), we could only observe a decrease of cells in the S phase after 48 h incubation with 2e. In parallel, the tendency to increase the percentage of cells in G0/G1 phase was observed. Cisplatin used as a control of the test increased cells percentage in the G2M phase. Analysis of dead cells (subG1) showed a significant increase in dead cells caused by 2f used in higher concentrations. Next, we decided to analyze apoptotic and necrotic cells using Annexin V/PI staining (Figure 2E–G), as well as the activity of caspase 3/7 in the treated cells (Figure 2H). Compound 2b increased the level of early apoptotic cells with an increase of caspase 3/7 activity. Compound 2f increased the percentage of necrotic cells (but also the tendency to increase the level of apoptotic cells: early and late, was observed) and also increased the activity of caspase 3/7. In the case of compound 2e, an increased percentage of late apoptotic cells was accompanied by an increase of caspase 3/7 activity. The drop of the percentage of cells with high mitochondrial membrane potential (ΔΨ) was observed in MV4-11 cells incubated with compound 2f and 2e (Figure 2I). For labeling autophagic vacuoles, two techniques with dansyl cadaverin and with acridine orange were used. In both methods, compound 2f increased the level of acidic autophagic vacuoles (Figure 2J,K).

## 4. Conclusions

In this study, we prepared aminophosphonic acids and their derivatives based on octahydroquinoxalin-2(1*H*)-one scaffold via Pudovik reaction. The syntheses proceeded efficiently giving stable products in case of all types of *H*-phosphonates, phosphine oxides and phosphite used. Since no significant differences of antiproliferative activities for a diastereomeric mixture (2f) and a single epimer (2g) were observed, further studies were conducted for mixtures of both stereoisomers of each compound.

Compound 2b, which was found to be the most active in proliferation inhibition of the MV4-11 cells, induces apoptosis of these cells with an increased caspase 3/7 activity. Compound 2f, which inhibited the proliferation of all neoplastic cell lines, tends to decrease the percentage of cells in the G2M phase and increases the percentage of dead cells, including cells undergoing necrosis. This compound increases the activity of caspases 3/7 and reduces the mitochondrial potential of cells. A long-lasting drop or rise of ΔΨ from control levels may induce a loss of cell viability. The higher is the level of intracellular ATP, the more stable are the ΔΨ values, making ATP a compound buffering mitochondrial ΔΨ [42]. However, compound 2f can also enhance autophagy. This phenomenon may not be beneficial from the point of view of cancer therapies, although there are different opinions, as well as it may depend on specific mechanisms of action of studied compounds [43]. Derivative 2e reduces the percentage of cells in the S phase of the cell cycle, increases the percentage of cells in late apoptosis and necrotic cells, increases caspase 3/7 activity, and reduces mitochondrial potential, so it works as a pro-apoptotic agent. A similar mechanism of action was reported by Huang’s group in the paper on the synthesis of potential anticancer candidates for the use in the therapy of ovarian cancer cells [25]. The tested compounds, with the emphasis put on 2b, 2e and 2f, could be used as promising scaffolds in further antiproliferative drug design.

## Figures and Tables

**Table 1:** The IR absorption of important functional groups.

Group	IR Absorption (KBr), cm^−1^									
	1	2a	2b	2c	2d	2e	2f	2g	3a	3b
**C=N** stretch (imine)	1622	-	-	-	-	-	-	-	-	-
**C=O** stretch (lactam)	1665	1666	1668	1668	1668	1692	1676	1675	1668	1646
**N–H** stretch (lactam)	3184	3192	3187	3169	3209	3197	3204	3204	3419	3369
**N–C** stretch	-	1416	1449	1419	1439	1437	1448	1448	1453	1447
**P=O** stretch	-	1251	1263	1241	1213	1166	1256	1256	1346	1349
**P–O–C** stretch (phosphonates)	-	1066	1191	1045	1036	-	1040	1040	-	-
**P–Ar** stretch	-	-	-	-	1122	1119	-	-		
P**–O(H)** stretch (phosphonic acids)	-	-	-	-	-	-	-	-	1068, 932	1087, 909

**Table 2:**
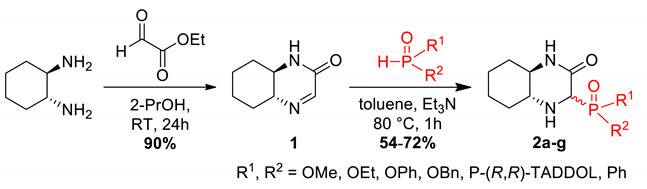
Synthesis of aminophosphonates and phosphine oxides.

No	R^1^	R^2^	Yield, %	*dr* (*R*:*S*)
2a	OMe	OMe	72	56:44
2b	OPh	OPh	69	50:50
2c	OBn	OBn	69	50:50
2d	OEt	Ph	54	50:50
2e	Ph	Ph	64	55:45
2f	P-(*R*,*R*)-TADDOL		72	60:40
2g ^A^	P-(*R*,*R*)-TADDOL		36	97:3

^A^ single epimer obtained by the crystallization of 2f.

**Table 3:** Antiproliferative activity of aminophosphonates and phosphine oxides towards the MV4-11 leukemia cell line.

Compound	Cell Line/IC50 (µg/mL)				
	MV4-11	A549	LoVo	MCF-7	BALB/3T3
1	4.69 ± 1.71	19.69 ± 8.34	5.49 ± 0.44	24.07 ± 7.88	3.66 ± 2.93
2a	2.96 ± 0.42	n.a.	n.a.	n.a.	n.a.
2b	0.21 ± 0.13	n.a.	n.a.	n.a.	60.88 ± 36.80
2c	1.91 ± 1.31	n.a.	n.a.	70.62 ± 10.62	33.11 ± 1.86
2d	n.a.	n.t.	n.t.	n.t.	n.t.
2e	3.83 ± 3.76	n.a.	n.a.	n.a.	n.a.
2f	3.18 ± 0.11	4.41 ± 0.21	3.49 ± 0.36	5.10 ± 1.17	15.23 ± 10.44
2g	3.82 ± 2.42	4.63 ± 0.47	3.55 ± 0.35	18.92 ± 14.01	22.07 ± 11.50
3a	0.45 ± 0.25	n.a.	n.a.	n.a.	n.a.
3b	1.25 ± 0.91	n.a.	n.a.	n.a.	n.a.
Cisplatin	0.46 ± 0.26	1.94 ± 0.56	1.73 ± 0.67	1.9 ± 0.6	1.62 ± 0.69

n.a. not active in the range of concentrations used (0.1–100 µg/mL); n.t. not tested.

## Supplementary Materials

The following are available online at https://www.mdpi.com/1996-1944/13/10/2393/s1, Figure S1. (a) ^1^H NMR and (b) ^13^C NMR spectra of (1R,6R)-3-oxo-2,5-diazabicyclo[4.4.0]dec-4-ene 1. Figure S2. (a) ^1^H NMR, (b) ^13^C NMR and (c) ^31^P NMR spectra of dimethyl-[(1R,6R)-3-oxo-2,5-diazabicyclo[4.4.0]dec-4-yl]phosphonate 2a. Figure S3. (a) ^1^H NMR, (b) ^13^C NMR and (c) ^31^P NMR spectra of diphenyl-[(1R,6R)-3-oxo-2,5-diazabicyclo[4.4.0]dec-4-yl]phosphonate 2b. Figure S4. (a) ^1^H NMR, (b) ^13^C NMR and (c) ^31^P NMR spectra of dibenzyl-[(1R,6R)-3-oxo-2,5-diazabicyclo[4.4.0]dec-4-yl]phosphonate 2c. Figure S5. (a) ^1^H NMR, (b) ^13^C NMR and (c) ^31^P NMR spectra of ethyl-[(1*R*,6*R*)-3-oxo-2,5-diazabicyclo[4.4.0]dec-4-yl](phenyl)phosphinate 2d. Figure S6. (a) ^1^H NMR, (b) ^13^C NMR and (c) ^31^P NMR spectra of (1*R*,6*R*)-3-oxo-4-(diphenylphosphoryl)-2,5-diazabicyclo[4.4.0]decan 2e. Figure S7. (a) ^1^H NMR, (b) ^13^C NMR and (c) ^31^P NMR spectra of (4-((3a*R*,8a*R*)-2,2-dimethyl-6-oxido-4,4,8,8-tetraphenyltetrahydro-[1,3]dioxolo[4,5-e][1,3,2]dioxaphosphepin-6-yl))-(1*R*,6*R*)-3-oxo-2,5-diazabicyclo[4.4.0]decan 2f. Figure S8. (a) ^1^H NMR, (b) ^13^C NMR and (c) ^31^P NMR spectra of (4-((3a*R*,8a*R*)-2,2-dimethyl-6-oxido-4,4,8,8-tetraphenyltetrahydro-[1,3]dioxolo[4,5-e][1,3,2]dioxaphosphepin-6-yl))-(1*R*,6*R*)-3-oxo-2,5-diazabicyclo[4.4.0]decan 2g. Figure S9. (a) ^1^H NMR, (b) ^13^C NMR and (c) ^31^P NMR spectra of [(1*R*,6*R*)-3-oxo-2,5-diazabicyclo[4.4.0]dec-4-yl]-phosphonic acid 3a. Figure S10. (a) 1H NMR, (b) 13C NMR and (c) 31P NMR spectra of [4-phenyl-(1R,6R)-3-oxo-2,5-diazabicyclo[4.4.0]dec-4-yl]-phosphonic acid 3b.
