# Supplementary material for: Octahydroquinoxalin-2(1H)-One-Based Aminophosphonic Acids and Their Derivatives—Biological Activity towards Cancer Cells
Source: Materials (Basel). 2020 May 22;13(10):2393. doi: 10.3390/ma13102393 (PMC7287629; doi:10.3390/ma13102393)
Supplement: Supplementary file 1 [file materials-13-02393-s001.pdf]

materials

Supporting Information

Octahydroquinoxalin-2(1*H*)-One-Based Aminophosphonic Acids and Their Derivatives—Biological Activity Towards Cancer Cells

Jakub Iwanejko [1], Elżbieta Wojaczyńska [1,*], Eliza Turlej [2], Magdalena Maciejewska [2] and Joanna Wietrzyk [2]

[1] Faculty of Chemistry, Wrocław University of Science and Technology, Wybrzeże Wyspiańskiego 27, 50-370 Wrocław, Poland; jakub.iwanejko@pwr.edu.pl

[2] Department of Experimental Oncology, Hirszfeld Institute of Immunology and Experimental Therapy, Polish Academy of Sciences, Rudolfa Weigla 12, 53-114 Wrocław, Poland; eliza.turlej@hirszfeld.pl (E.T.); magdalena.maciejewska@hirszfeld.pl (M.M.); joanna.wietrzyk@hirszfeld.pl (J.W.)

* Correspondence: elzbieta.wojaczynska@pwr.edu.pl; Tel.: +48-71-3202410

Contents

1. NMR Spectra 2

Figure S1. (**a**) ^{1}H NMR and (**b**) ^{13}C NMR spectra of (1*R*,6*R*)-3-oxo-2,5-diazabicyclo[4.4.0]dec-4-ene 1. 2

Figure S2. (**a**) ^{1}H NMR, (**b**) ^{13}C NMR and (**c**) ^{31}P NMR spectra of dimethyl-[(1*R*,6*R*)-3-oxo-2,5-diazabicyclo[4.4.0]dec-4-yl]phosphonate 2a. 4

Figure S3. (**a**) ^{1}H NMR, (**b**) ^{13}C NMR and (**c**) ^{31}P NMR spectra of diphenyl-[(1*R*,6*R*)-3-oxo-2,5-diazabicyclo[4.4.0]dec-4-yl]phosphonate 2b. 5

Figure S4. (**a**) ^{1}H NMR, (**b**) ^{13}C NMR and (**c**) ^{31}P NMR spectra of dibenzyl-[(1*R*,6*R*)-3-oxo-2,5-diazabicyclo[4.4.0]dec-4-yl]phosphonate 2c. 7

Figure S5. (**a**) ^{1}H NMR, (**b**) ^{13}C NMR and (**c**) ^{31}P NMR spectra of ethyl-[(1*R*,6*R*)-3-oxo-2,5-diazabicyclo[4.4.0]dec-4-yl](phenyl)phosphinate 2d. 8

Figure S6. (**a**) ^{1}H NMR, (**b**) ^{13}C NMR and (**c**) ^{31}P NMR spectra of (1*R*,6*R*)-3-oxo-4-(diphenylphosphoryl)-2,5-diazabicyclo[4.4.0]decan 2e. 10

Figure S7. (**a**) ^{1}H NMR, (**b**) ^{13}C NMR and (**c**) ^{31}P NMR spectra of (4-((3a*R*,8a*R*)-2,2-dimethyl-6-oxido-4,4,8,8-tetraphenyltetrahydro-[1,3]dioxolo[4,5-e][1,3,2]dioxaphosphepin-6-yl))-(1*R*,6*R*)-3-oxo-2,5-diazabicyclo[4.4.0]decan 2f. 11

Figure S8. (**a**) ^{1}H NMR, (**b**) ^{13}C NMR and (**c**) ^{31}P NMR spectra of (4-((3a*R*,8a*R*)-2,2-dimethyl-6-oxido-4,4,8,8-tetraphenyltetrahydro-[1,3]dioxolo[4,5-e][1,3,2]dioxaphosphepin-6-yl))-(1*R*,6*R*)-3-oxo-2,5-diazabicyclo[4.4.0]decan 2g. 13

Figure S9. (**a**) ^{1}H NMR, (**b**) ^{13}C NMR and (**c**) ^{31}P NMR spectra of [(1*R*,6*R*)-3-oxo-2,5-diazabicyclo[4.4.0]dec-4-yl]-phosphonic acid 3a. 14

Figure S10. (**a**) ^{1}H NMR, (**b**) ^{13}C NMR and (**c**) ^{31}P NMR spectra of [4-phenyl-(1*R*,6*R*)-3-oxo-2,5-diazabicyclo[4.4.0]dec-4-yl]-phosphonic acid 3b. 16

1. NMR Spectra

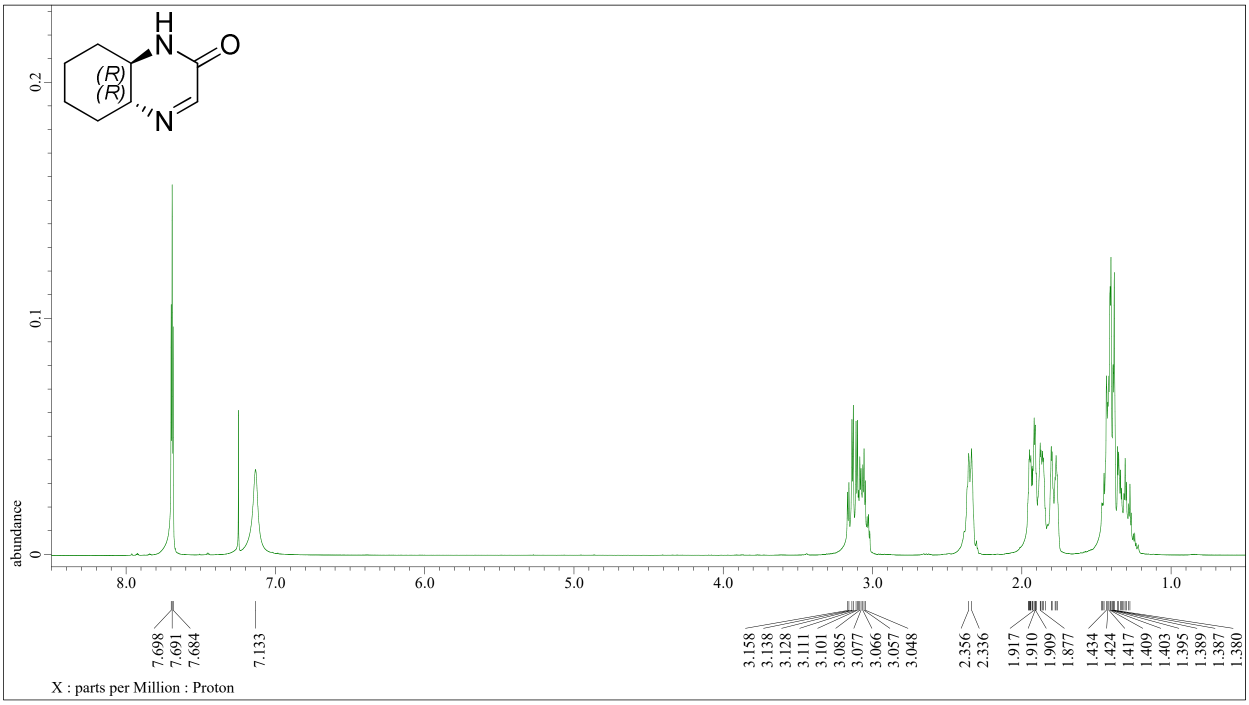

(**a**)

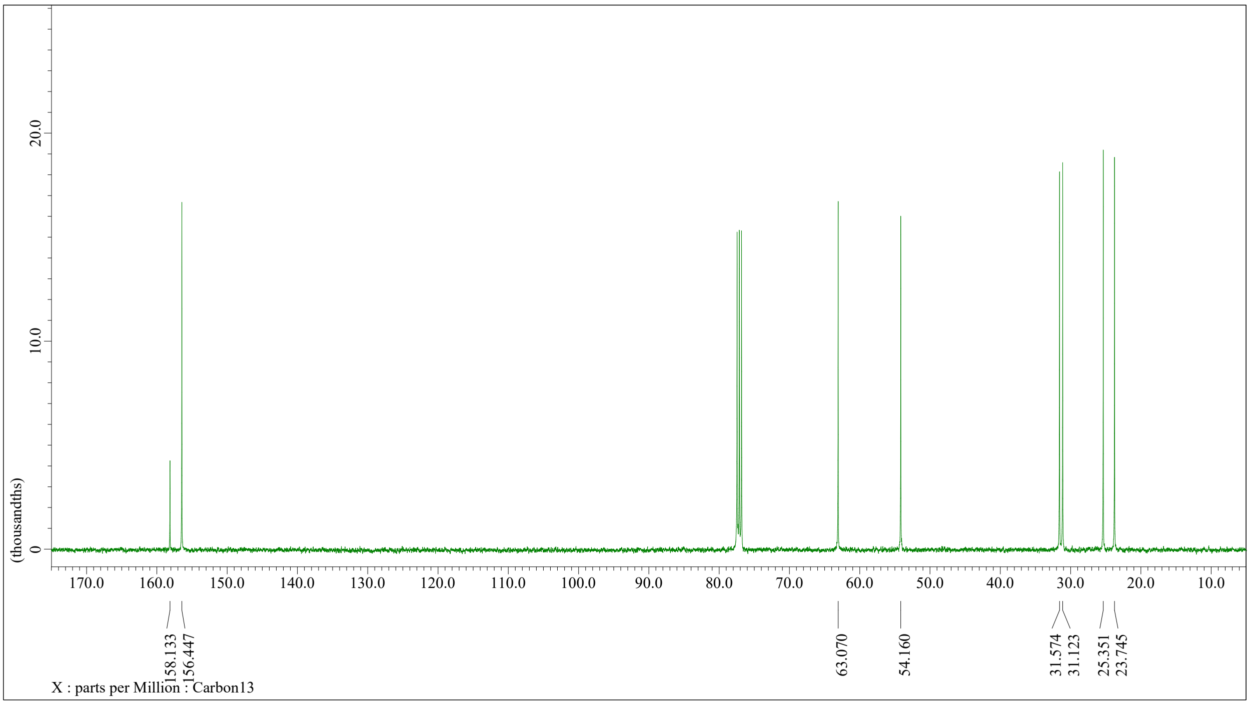

(**b**)

Figure S1. (**a**) ^{1}H NMR and (**b**) ^{13}C NMR spectra of (1*R*,6*R*)-3-oxo-2,5-diazabicyclo[4.4.0]dec-4-ene 1.

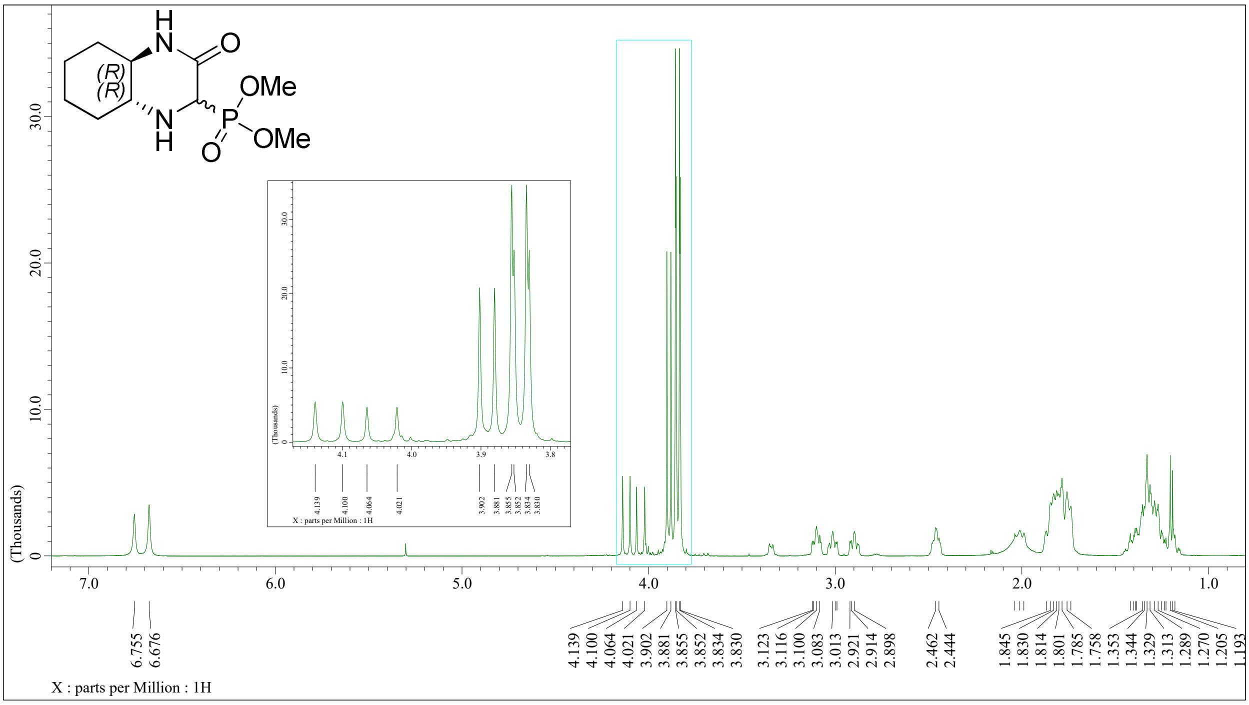

(R)
(R)
N
H
O
OMe
P
OMe
O
(Thousands)
0
10.0
20.0
30.0
7.0
6.0
5.0
4.0
3.0
2.0
1.0
6.755
6.676
4.139
4.100
4.064
4.021
3.902
3.881
3.855
3.852
3.834
3.830
3.123
3.116
3.100
3.083
3.013
2.921
2.914
2.898
2.462
2.444
1.845
1.830
1.814
1.801
1.785
1.758
1.353
1.344
1.329
1.313
1.289
1.270
1.205
1.193
X : parts per Million : 1H

(**a**)

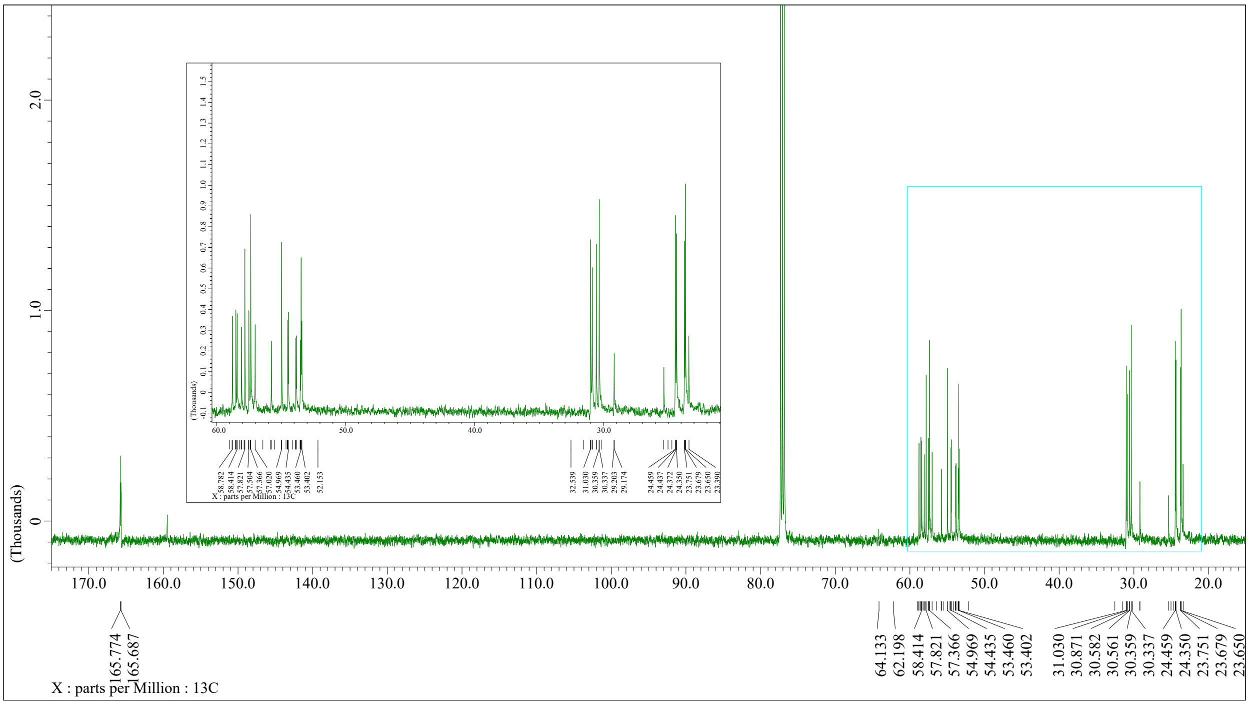

(Thousands)
0
1.0
2.0
170.0
160.0
150.0
140.0
130.0
120.0
110.0
100.0
90.0
80.0
70.0
60.0
50.0
40.0
30.0
20.0
165.774
165.687
64.133
62.198
58.414
57.821
57.366
54.969
54.435
53.460
53.402
31.030
30.871
30.582
30.561
30.359
30.337
24.459
24.350
23.751
23.679
23.650
X : parts per Million : 13C

(**b**)

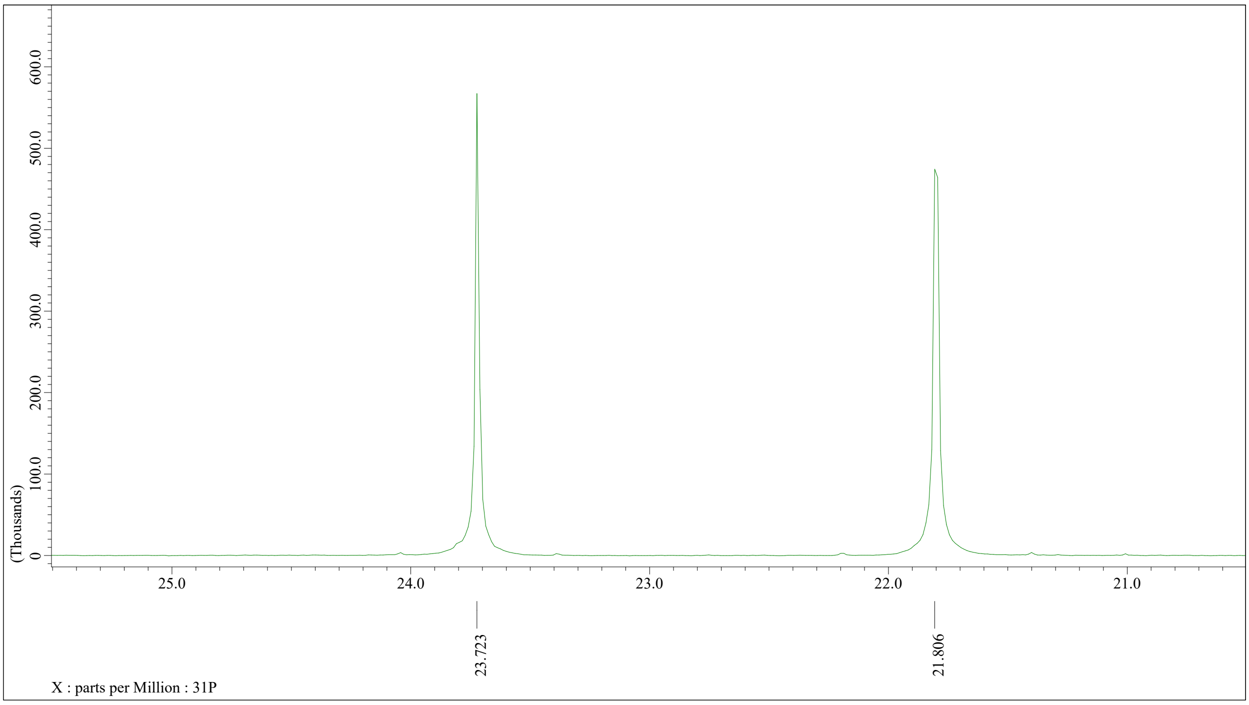

(**c**)

Figure S2. (**a**) ^{1}H NMR, (**b**) ^{13}C NMR and (**c**) ^{31}P NMR spectra of dimethyl-[(1*R*,6*R*)-3-oxo-2,5-diazabicyclo[4.4.0]dec-4-yl]phosphonate 2a.

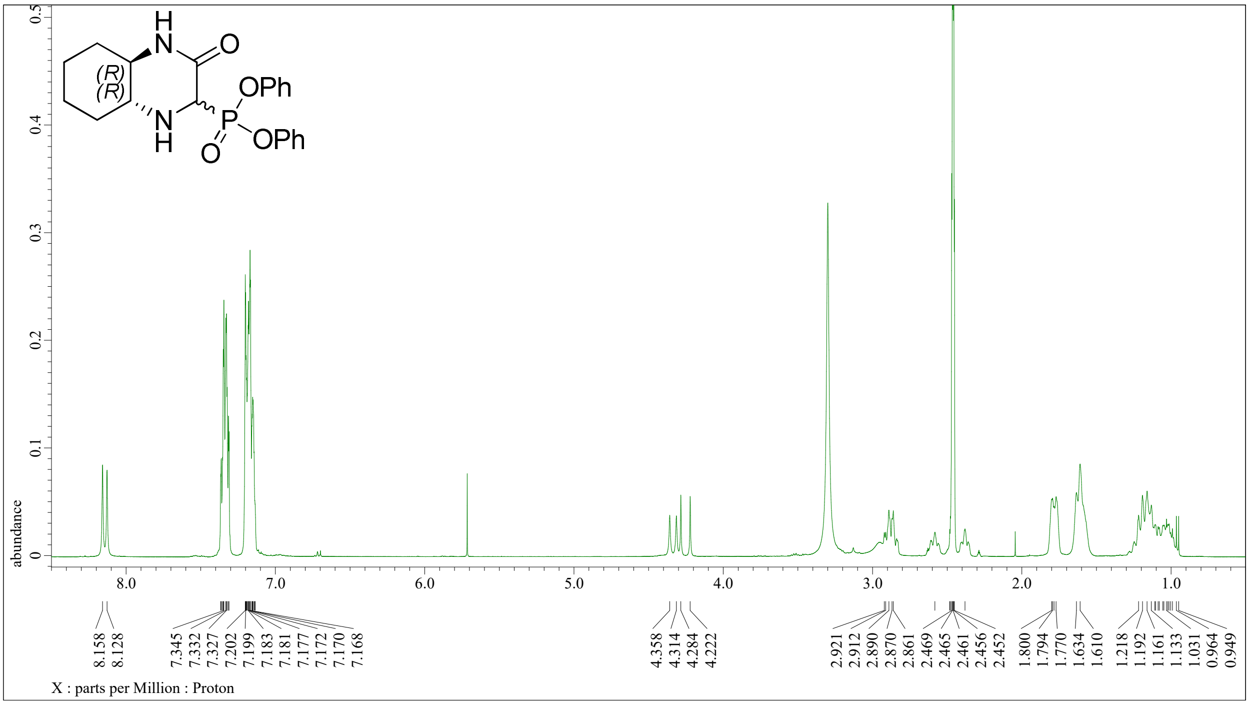

(**a**)

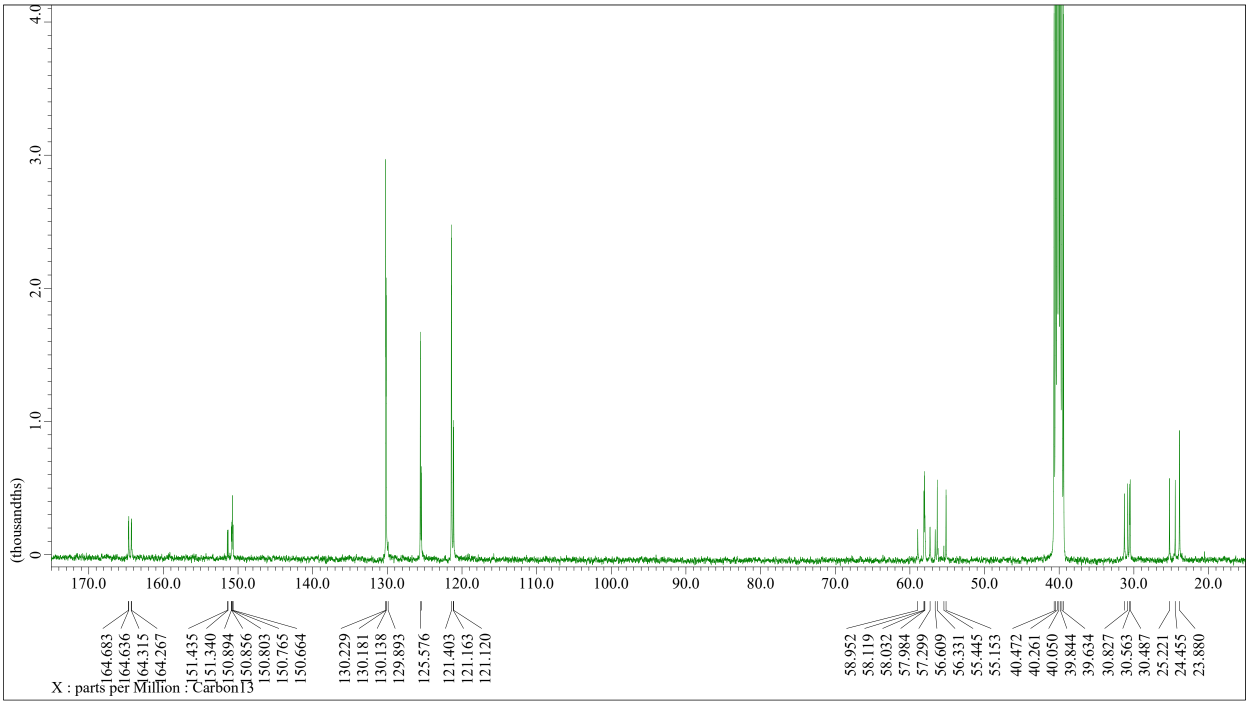

(**b**)

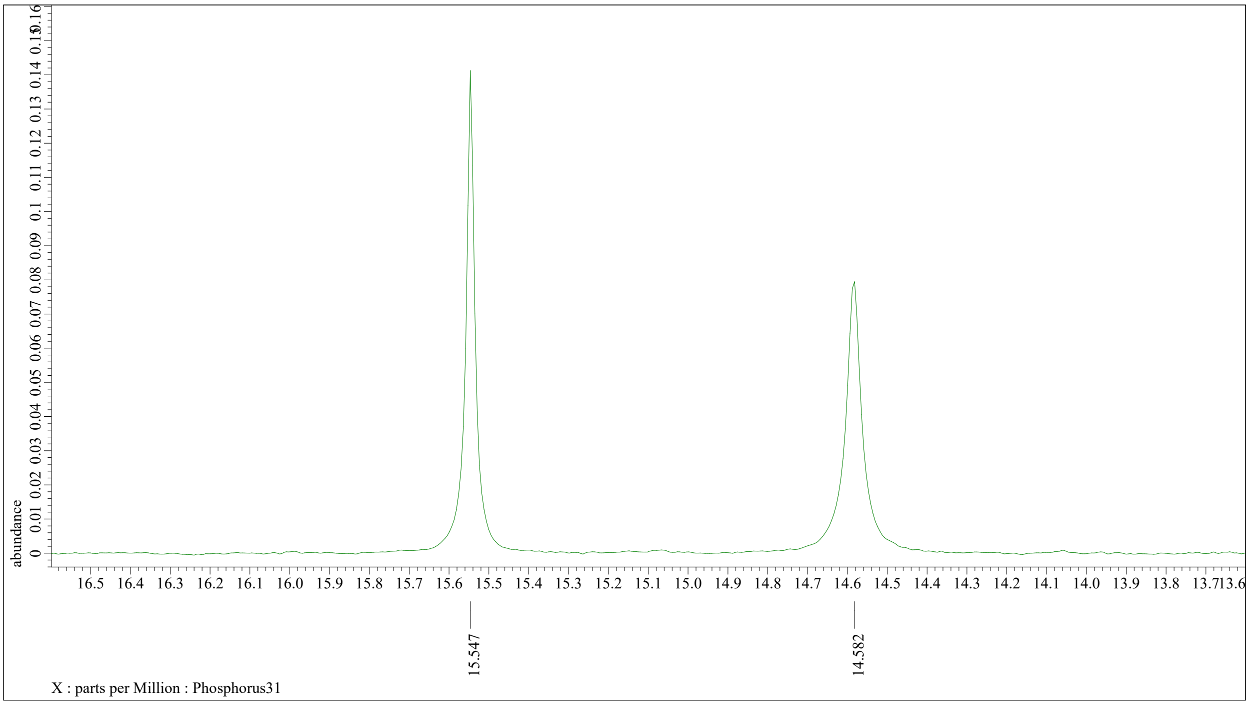

(**c**)

Figure S3. (**a**) ^{1}H NMR, (**b**) ^{13}C NMR and (**c**) ^{31}P NMR spectra of diphenyl-[(1*R*,6*R*)-3-oxo-2,5-diazabicyclo[4.4.0]dec-4-yl]phosphonate 2b.

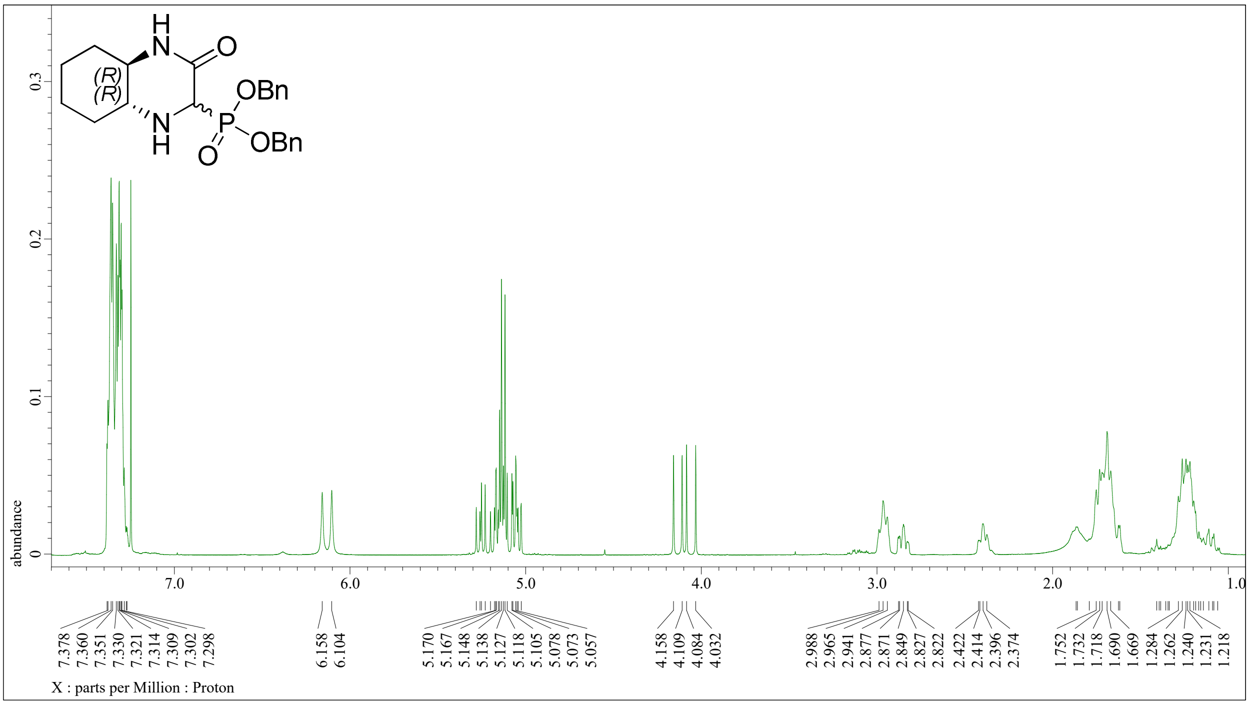

H
N
O
(R)
(R)
OBn
N
H
P
O
OBn
abundance
X : parts per Million : Proton
7.378
7.360
7.351
7.330
7.321
7.314
7.309
7.302
7.298
6.158
6.104
5.170
5.167
5.148
5.138
5.127
5.118
5.105
5.078
5.073
5.057
4.158
4.109
4.084
4.032
2.988
2.965
2.941
2.877
2.871
2.849
2.827
2.822
2.422
2.414
2.396
2.374
1.752
1.732
1.718
1.690
1.669
1.284
1.262
1.240
1.231
1.218

(**a**)

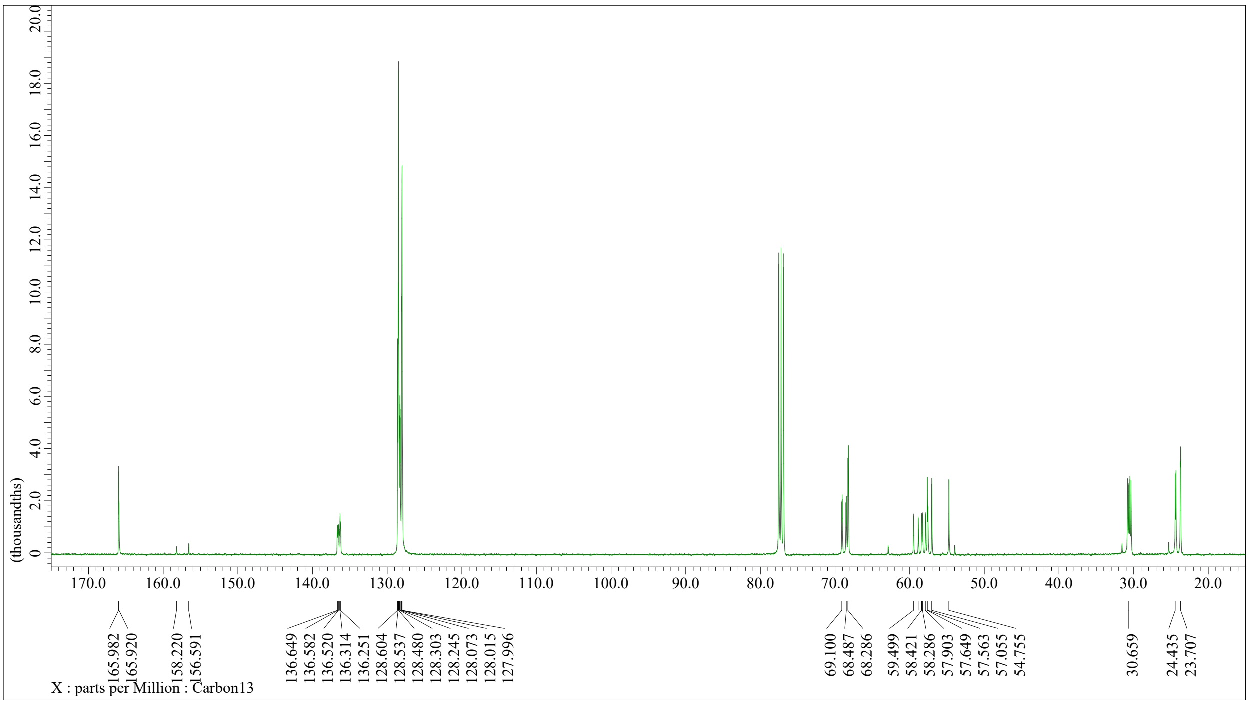

(thousandths)
X : parts per Million : Carbon13
165.982
165.920
158.220
156.591
136.649
136.582
136.520
136.314
136.251
128.604
128.537
128.480
128.303
128.245
128.073
128.015
127.996
69.100
68.487
68.286
59.499
58.421
58.286
57.903
57.649
57.563
57.055
54.755
30.659
24.435
23.707

(**b**)

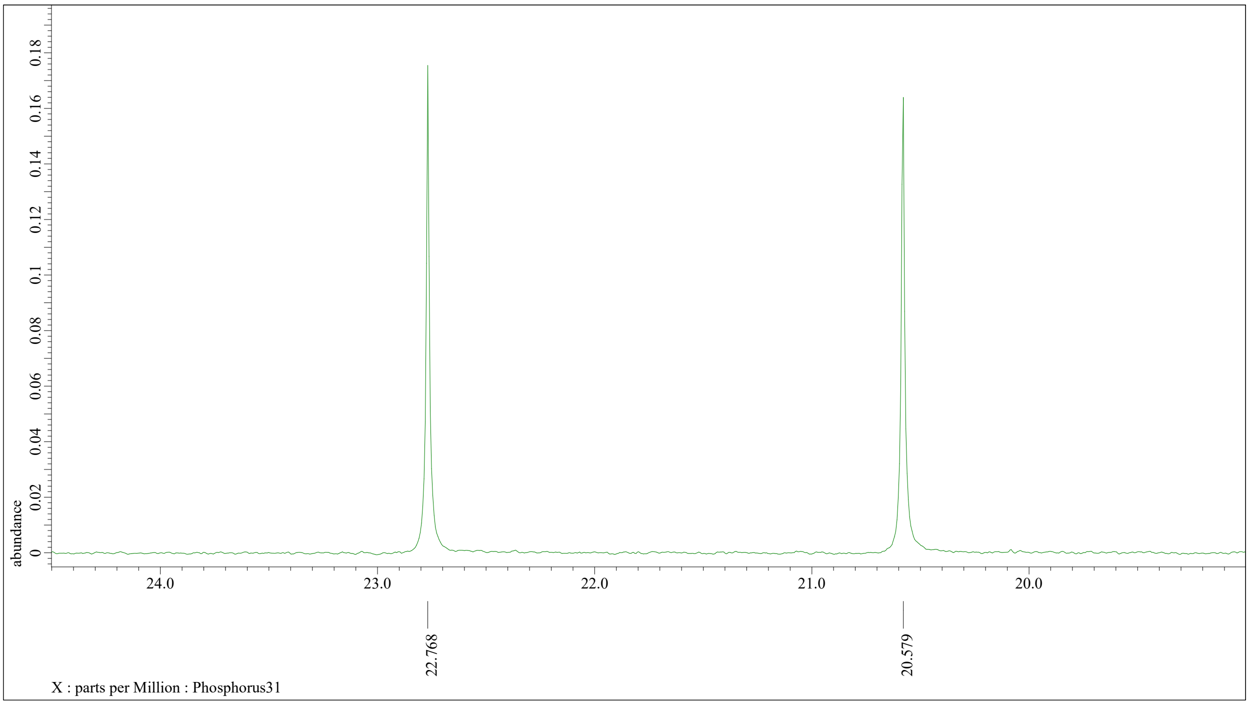

(**c**)

Figure S4. (**a**) ^{1}H NMR, (**b**) ^{13}C NMR and (**c**) ^{31}P NMR spectra of dibenzyl-[(1*R*,6*R*)-3-oxo-2,5-diazabicyclo[4.4.0]dec-4-yl]phosphonate 2c.

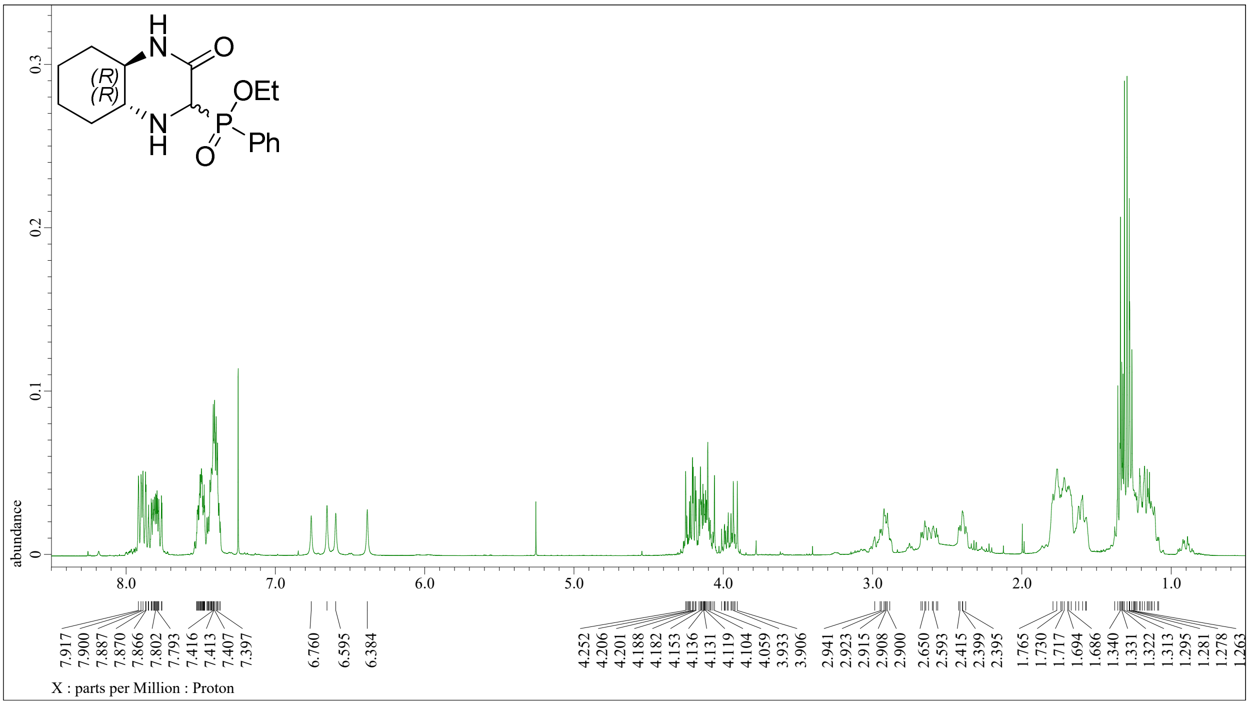

(**a**)

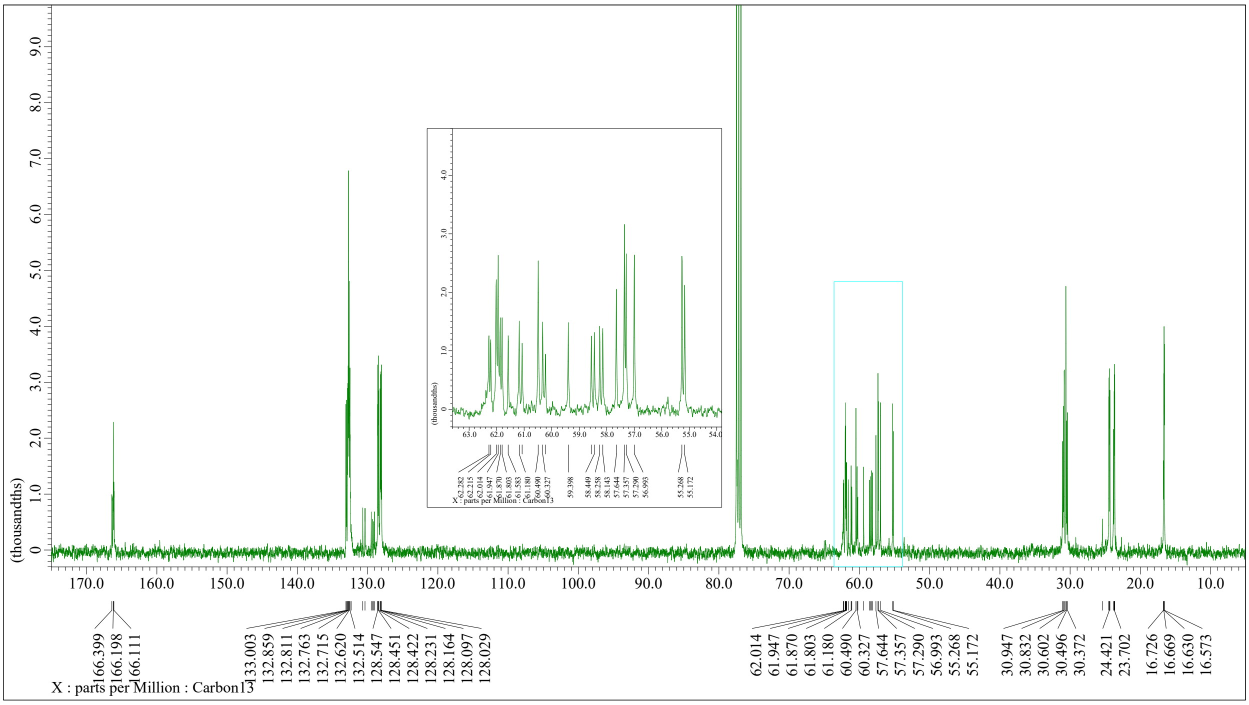

(**b**)

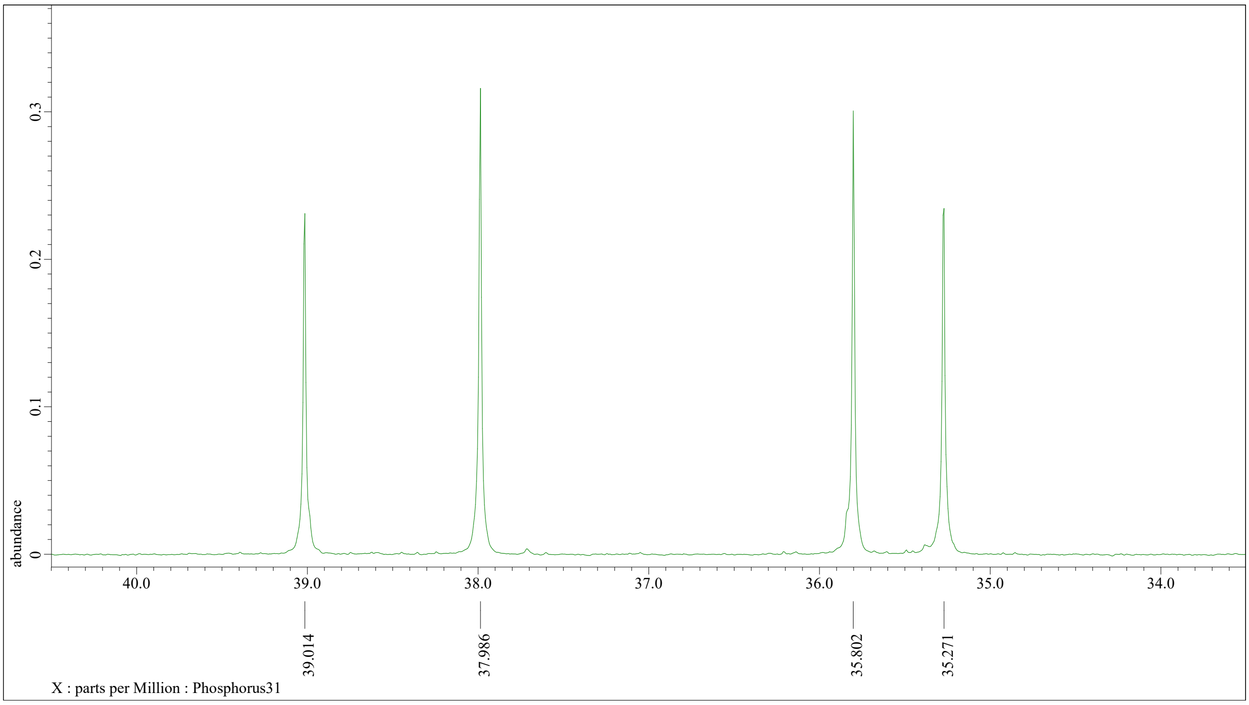

(**c**)

Figure S5. (**a**) ^{1}H NMR, (**b**) ^{13}C NMR and (**c**) ^{31}P NMR spectra of ethyl-[(1*R*,6*R*)-3-oxo-2,5-diazabicyclo[4.4.0]dec-4-yl](phenyl)phosphinate 2d.

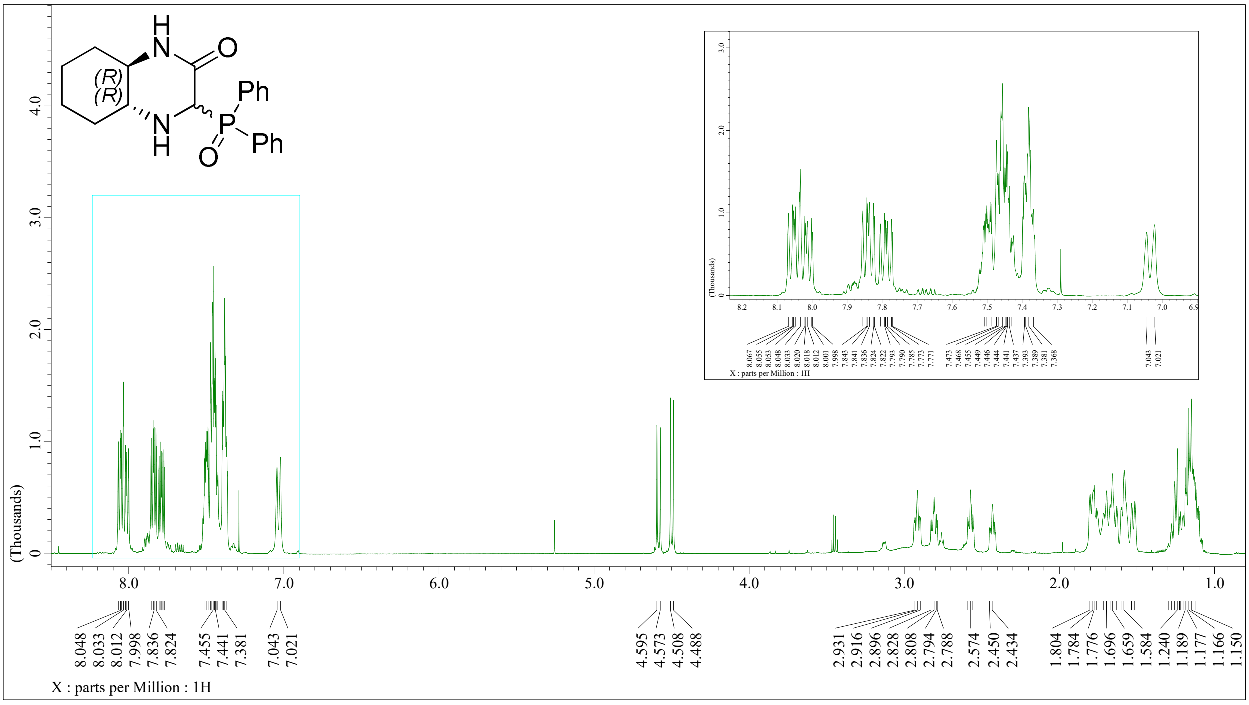
X : parts per Million : 1H
(Thousands)
8.048
8.033
8.012
7.998
7.836
7.824
7.455
7.441
7.381
7.043
7.021
4.595
4.573
4.508
4.488
2.931
2.916
2.896
2.828
2.808
2.794
2.788
2.574
2.450
2.434
1.804
1.784
1.776
1.696
1.659
1.584
1.240
1.189
1.177
1.166
1.150

(**a**)

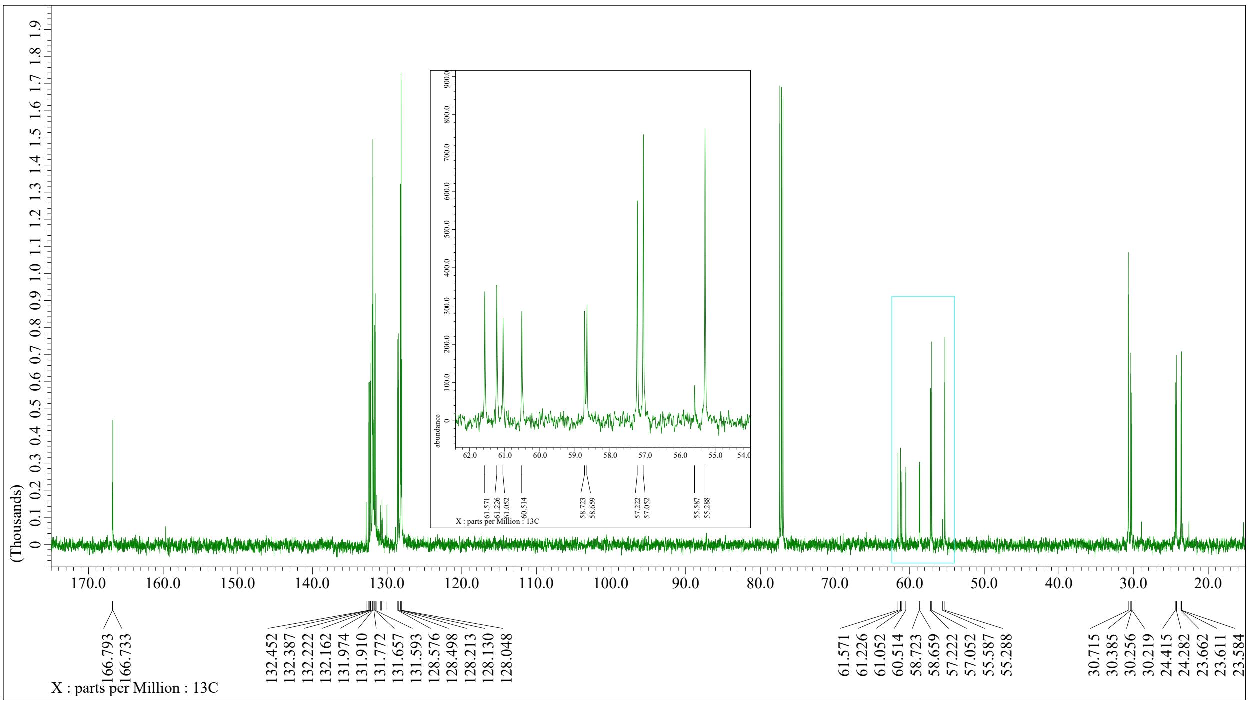
X : parts per Million : 13C
(Thousands)
166.793
166.733
132.452
132.387
132.222
132.162
131.974
131.910
131.772
131.657
131.593
128.576
128.498
128.213
128.130
128.048
61.571
61.226
61.052
60.514
58.723
58.659
57.222
57.052
55.587
55.288
30.715
30.385
30.256
30.219
24.415
24.282
23.662
23.611
23.584

(**b**)

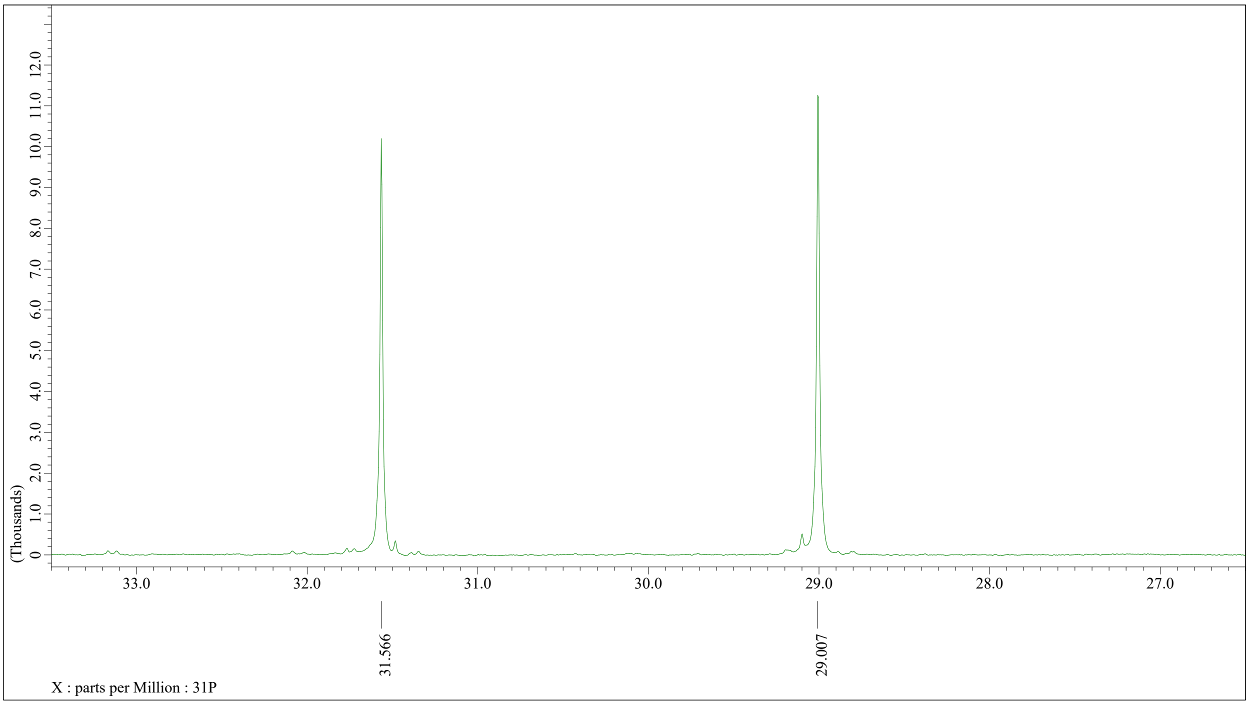

(**c**)

Figure S6. (**a**) ^{1}H NMR, (**b**) ^{13}C NMR and (**c**) ^{31}P NMR spectra of (1*R*,6*R*)-3-oxo-4-(diphenylphosphoryl)-2,5-diazabicyclo[4.4.0]decan 2e.

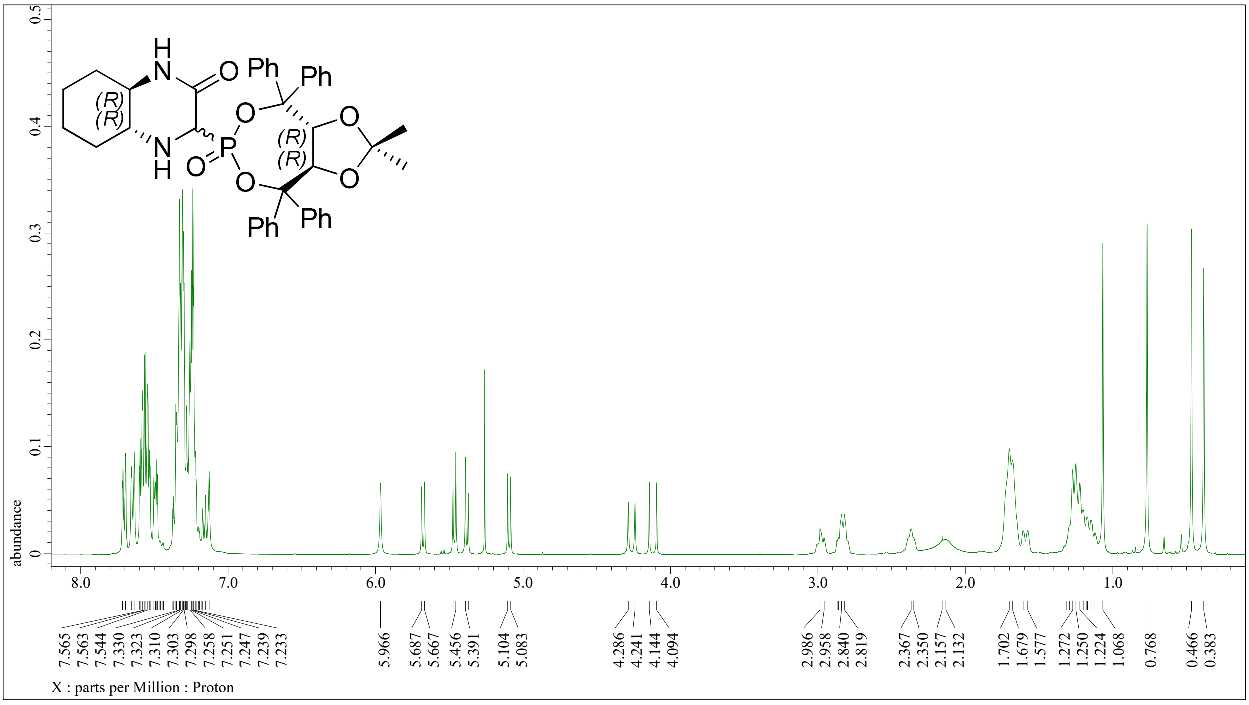

(**a**)

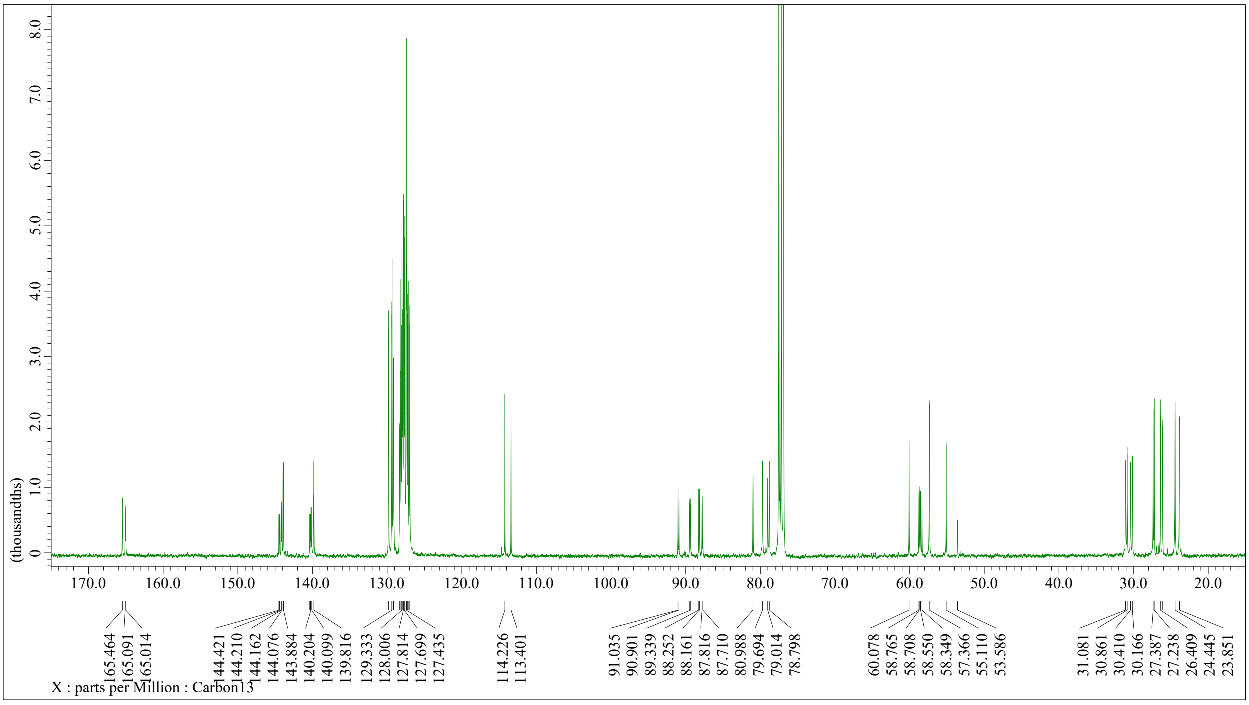

(**b**)

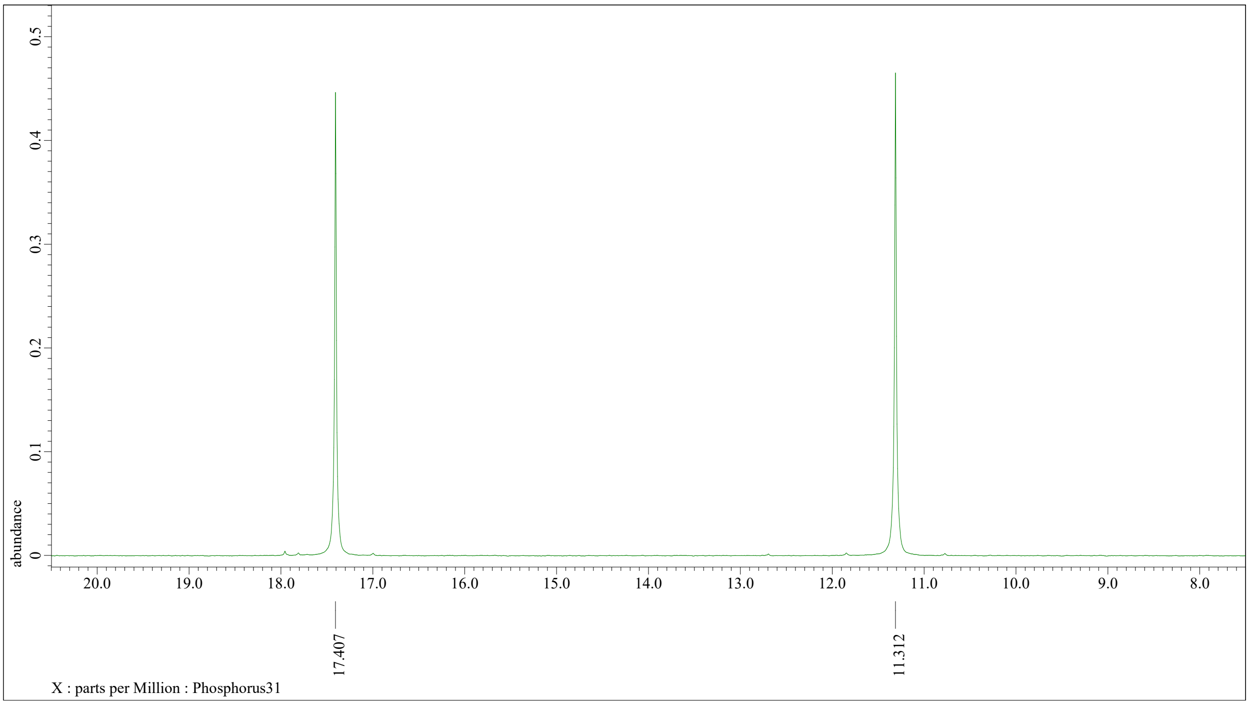

(**c**)

Figure 7. (**a**) ^{1}H NMR, (**b**) ^{13}C NMR and (**c**) ^{31}P NMR spectra of (4-((3a*R*,8a*R*)-2,2-dimethyl-6-oxido-4,4,8,8-tetraphenyltetrahydro-[1,3]dioxolo[4,5-e][1,3,2]dioxaphosphepin-6-yl))-(1*R*,6*R*)-3-oxo-2,5-diazabicyclo[4.4.0]decan 2f.

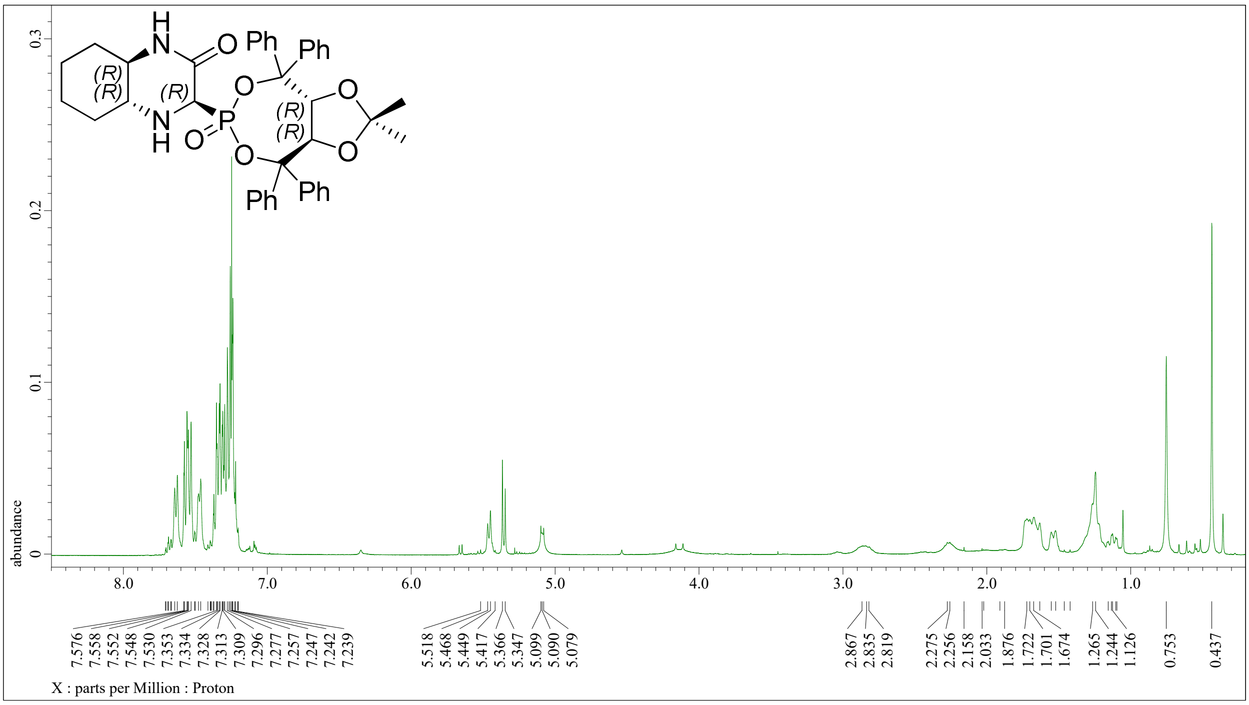

H
N
O
Ph
Ph
(R)
(R)
(R)
O
(R)
(R)
O
N
H
O
P
O
O
Ph
Ph
abundance
0.3
0.2
0.1
0
8.0
7.0
6.0
5.0
4.0
3.0
2.0
1.0
7.576 7.558 7.552 7.548 7.530 7.353 7.334 7.328 7.313 7.309 7.296 7.277 7.257 7.247 7.242 7.239
5.518 5.468 5.449 5.417 5.366 5.347 5.099 5.090 5.079
2.867 2.835 2.819
2.275 2.256 2.158 2.033 1.876 1.722 1.701 1.674
1.265 1.244 1.126
0.753
0.437
X : parts per Million : Proton

(**a**)

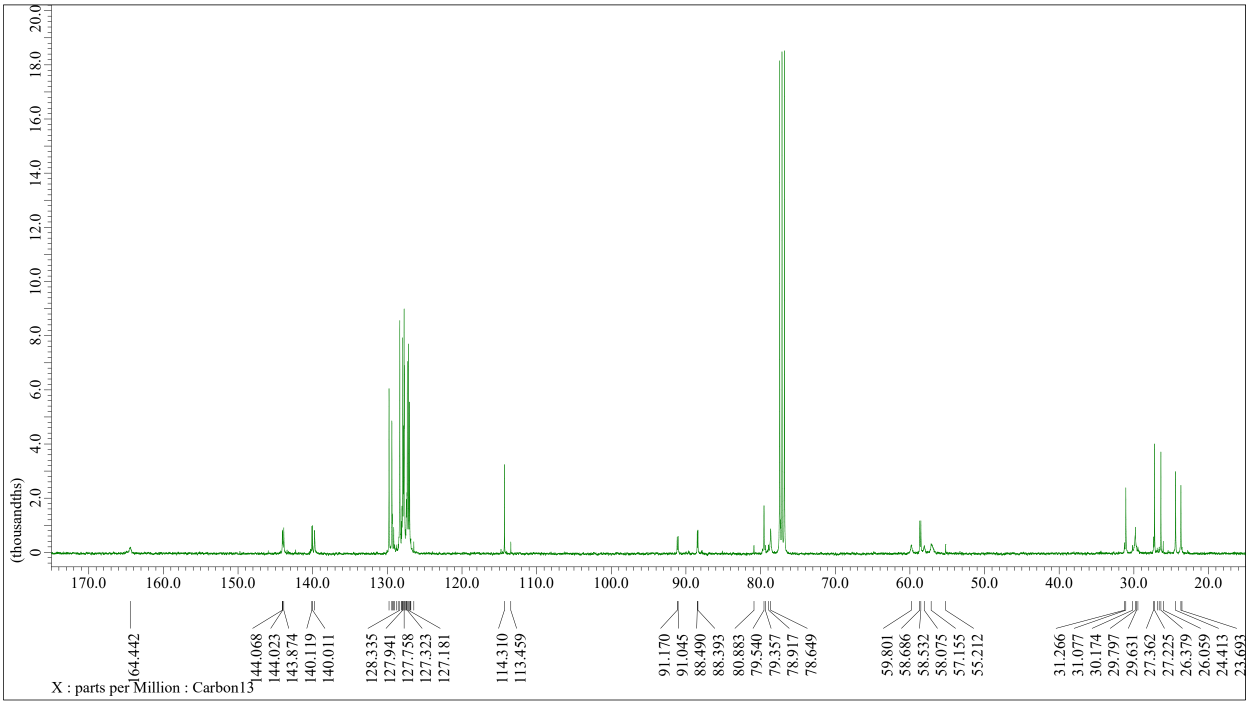

(thousandths)
20.0
18.0
16.0
14.0
12.0
10.0
8.0
6.0
4.0
2.0
0
170.0
160.0
150.0
140.0
130.0
120.0
110.0
100.0
90.0
80.0
70.0
60.0
50.0
40.0
30.0
20.0
164.442
144.068 144.023 143.874 140.119 140.011
128.335 127.941 127.758 127.323 127.181
114.310 113.459
91.170 91.045 88.490 88.393
80.883 79.540 79.357 78.917 78.649
59.801 58.686 58.532 58.075 57.155 55.212
31.266 31.077 30.174 29.797 29.631 27.362 27.225 26.379 26.059 24.413 23.693
X : parts per Million : Carbon13

(**b**)

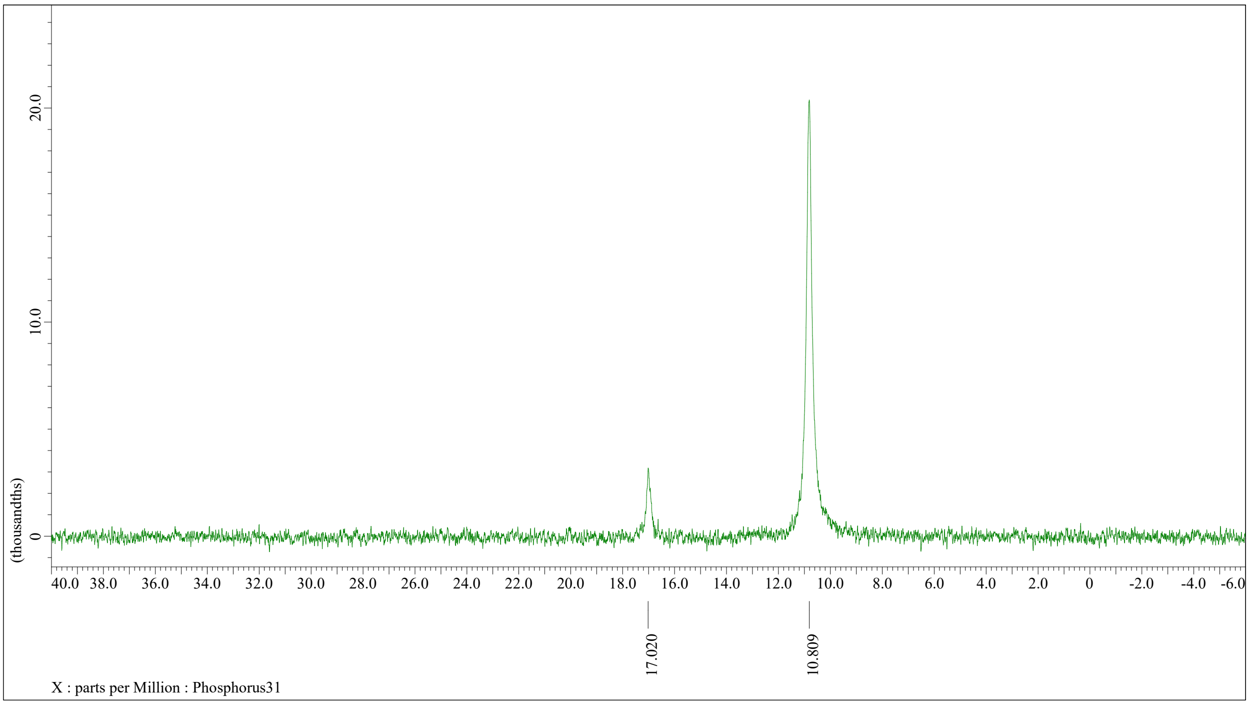

(**c**)

Figure S8. (**a**) ^{1}H NMR, (**b**) ^{13}C NMR and (**c**) ^{31}P NMR spectra of (4-((3a*R*,8a*R*)-2,2-dimethyl-6-oxido-4,4,8,8-tetraphenyltetrahydro-[1,3]dioxolo[4,5-e][1,3,2]dioxaphosphepin-6-yl))-(1*R*,6*R*)-3-oxo-2,5-diazabicyclo[4.4.0]decan 2g.

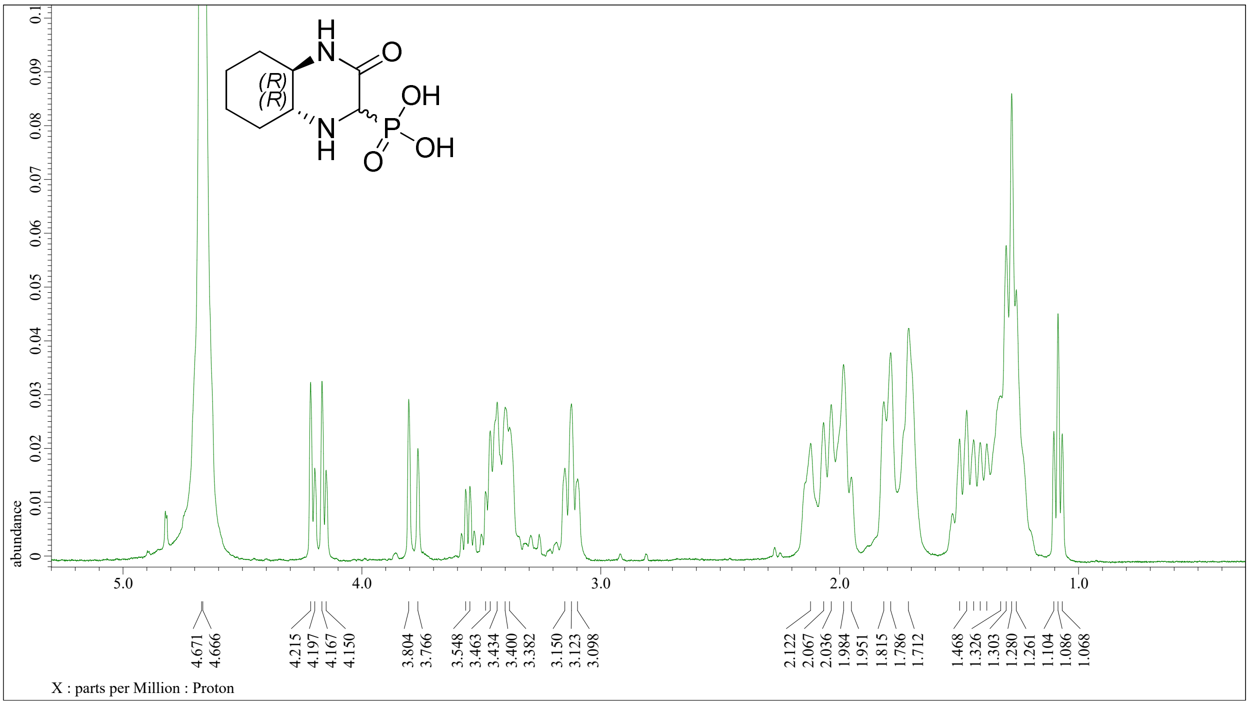

(**a**)

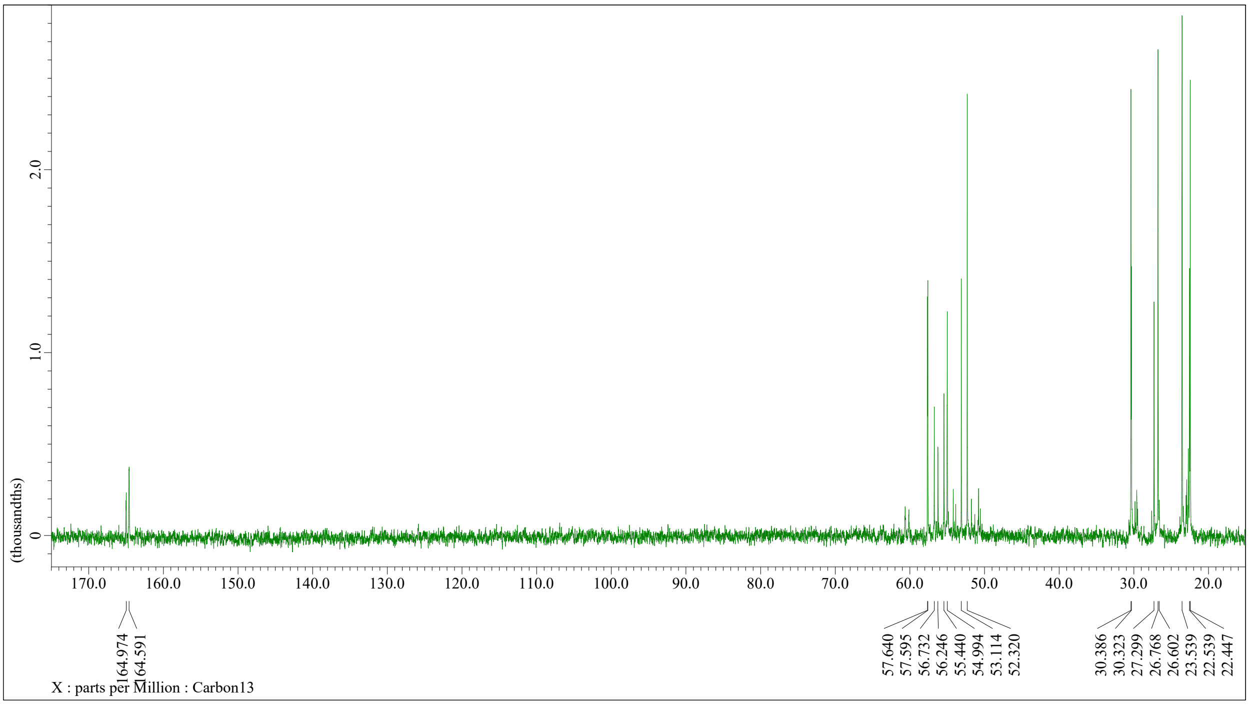

(**b**)

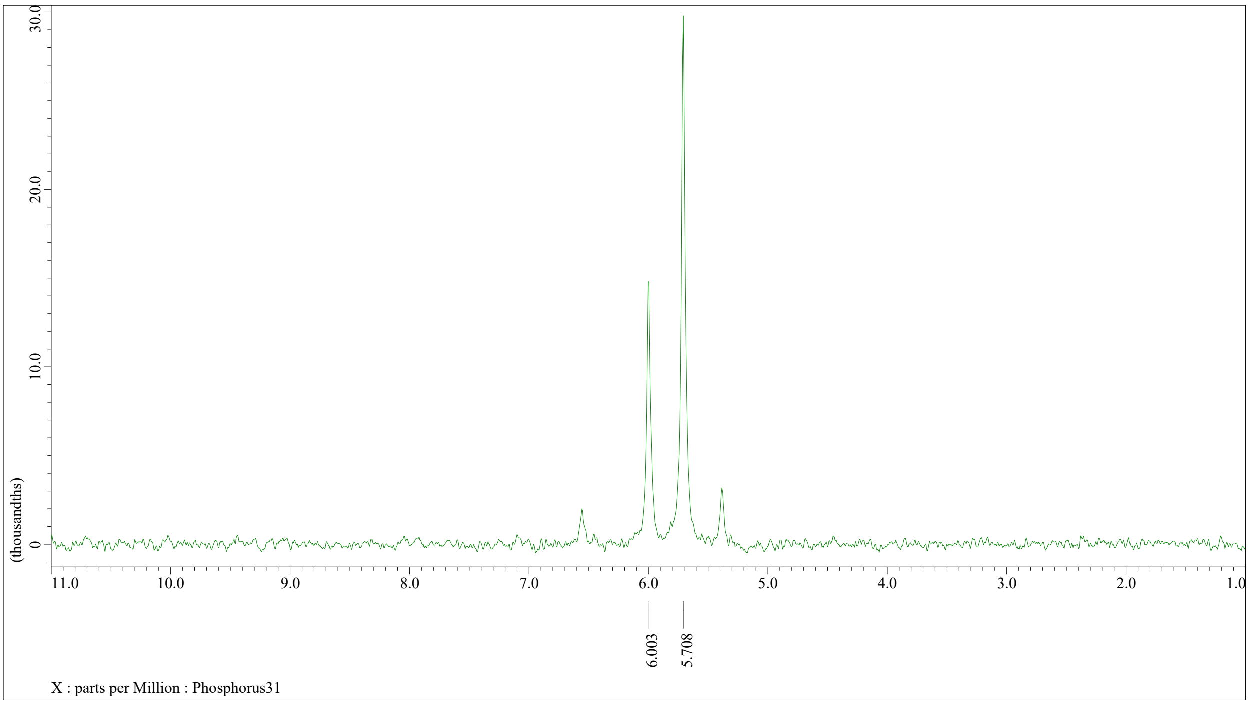

(**c**)

Figure S9. (**a**) ^{1}H NMR, (**b**) ^{13}C NMR and (**c**) ^{31}P NMR spectra of [(1*R*,6*R*)-3-oxo-2,5-diazabicyclo[4.4.0]dec-4-yl]-phosphonic acid 3a.

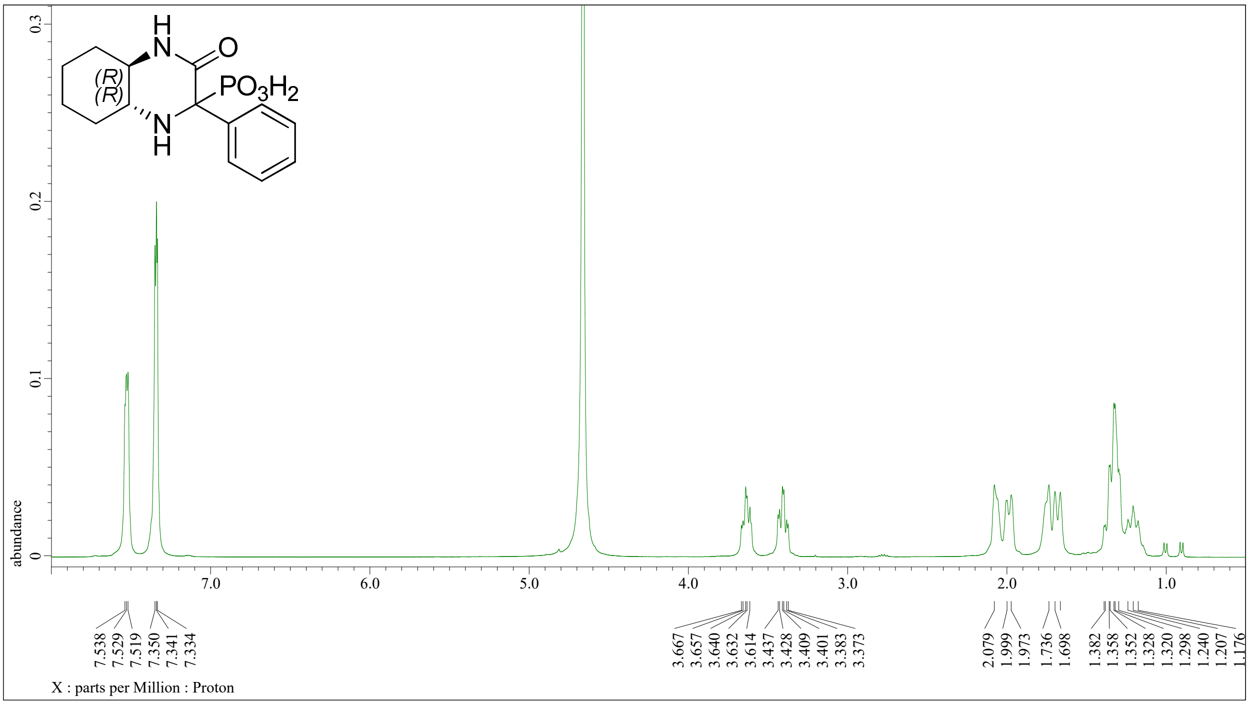

(R)
(R)
PO3H2
abundance
X : parts per Million : Proton
7.538 7.529 7.519 7.350 7.341 7.334
3.667 3.657 3.640 3.632 3.614 3.437 3.428 3.409 3.401 3.383 3.373
2.079 1.999 1.973 1.736 1.698
1.382 1.358 1.352 1.328 1.320 1.298 1.240 1.207 1.176

(**a**)

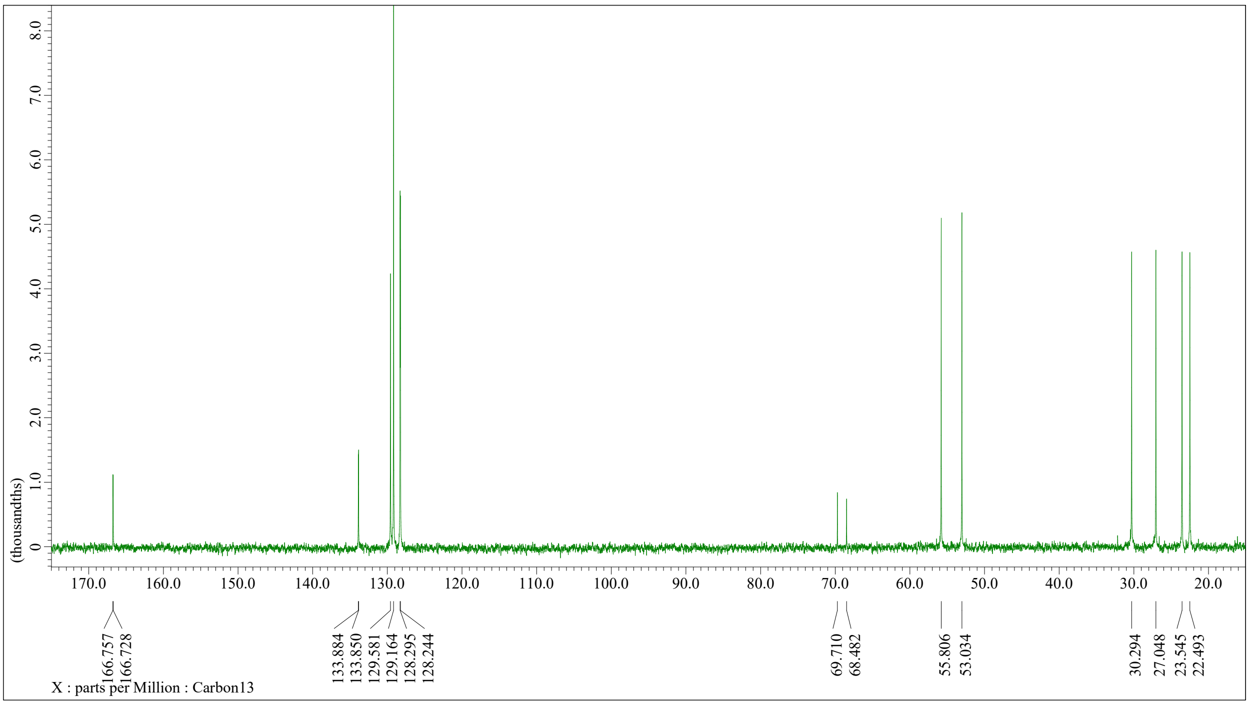

(thousandths)
X : parts per Million : Carbon13
166.757 166.728
133.884 133.850 129.581 129.164 128.295 128.244
69.710 68.482
55.806 53.034
30.294 27.048 23.545 22.493

(**b**)

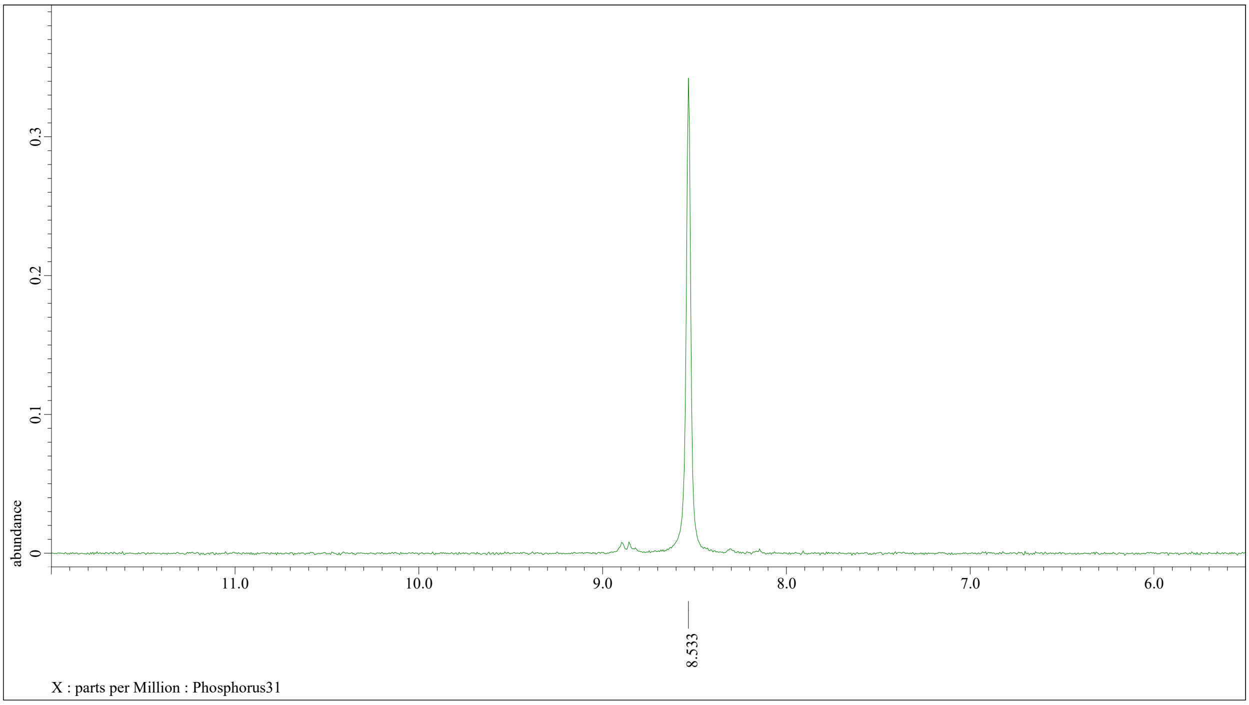

(**c**)

Figure S10. (**a**) ^{1}H NMR, (**b**) ^{13}C NMR and (**c**) ^{31}P NMR spectra of [4-phenyl-(1*R*,6*R*)-3-oxo-2,5-diazabicyclo[4.4.0]dec-4-yl]-phosphonic acid 3b.

© 2020 by the authors. Submitted for possible open access publication under the terms and conditions of the Creative Commons Attribution (CC BY) license (http://creativecommons.org/licenses/by/4.0/).
